# Comparative Analysis of Key Odorants and Aroma Characteristics in Hot-Pressed Yellow Horn (*Xanthoceras sorbifolia bunge*) Seed Oil Via Gas Chromatography–Ion Mobility Spectrometry and Gas Chromatography–Olfactory-Mass Spectrometry

**DOI:** 10.3390/foods12173174

**Published:** 2023-08-23

**Authors:** Hui Gao, Mengkai Liu, Lili Zheng, Tingting Zhang, Xiuliang Chang, He Liu, Sen Zhou, Zhiran Zhang, Shengxin Li, Jie Sun

**Affiliations:** 1 College of Life Sciences, Qingdao University, Qingdao 266071, China; gaochuqi01@163.com (H.G.); liumengkaikai@163.com (M.L.);; 2 National Engineering Research Centre for Intelligent Electrical Vehicle Power System (Qingdao), College of Mechanical & Electronic Engineering, Qingdao University, Qingdao 266071, China

**Keywords:** yellow horn seed oil, key odorants, gc-ims, gc-o-ms, multivariate statistical analysis

## Abstract

Volatile compounds (VOCs) present in the oil extracted from yellow horn seeds were first analyzed using GC-IMS and GC-O-MS at varying roasting temperatures. A total of 97 VOCs were detected using GC-IMS, while 77 were tentatively identified using GC-O-MS. Moreover, both methods allowed the identification of 24 VOCs, of which the type of aldehydes is the most abundant. Combining the results of GC-IMS, GC-O-MS, OAVs, and VIP, it was concluded that hexanal, 2,5-dimethylpyrazine, heptanal, 2-pentylfuran, 1-hexanol, and 1-octen-3-ol were the key aroma compounds. The PLS-DA and OPLS-DA models have demonstrated the ability to discriminate between different oil roasting temperatures with high accuracy. The roasting temperature of 160 °C was found to yield the highest content of main aroma substances, indicating its optimality for yellow horn seed oil production. These findings will prove beneficial for optimizing industrial production and enhancing oil aroma control.

## 1. Introduction

*Xanthoceras sorbifolia bunge* (yellow horn), a deciduous tree widely distributed in central and northern China, represents the sole species within the genus *Sapindaceae* [1]. The oil content of yellow horn seed is up to 55–65% [2], being considered a high-quality functional edible oil with an unsaturated fatty acid content of 85–93% [3,4,5], including linoleic (41.2%), oleic (42.3%) and nervonic acids (2–5%) [6]. Nervonic acid is capable of enhancing cognitive function and memory, as well as repairing damage to the central nervous system [7]. The production and consumption of yellow horn seed oil have been on the rise in recent years, owing to the growing recognition of its nutritional and functional properties by consumers. The seeds are roasted at an optimal temperature and time before being pressed to extract the oil, which boasts a robust aroma that is highly valued by consumers [8].

In the current process of plant oil production, raw materials are typically subjected to elevated temperatures of roasting prior to pressing to increase oil yield and enhance aroma [9]. Properly executed high-temperature roasting can enrich oils with aromatic substances through a series of reactions such as the Maillard reaction, lipid oxidation, Strecker degradation and caramelization [10], which generate a variety of aroma substances, including aldehydes, alcohols, and pyrazines [11].

Aldehydes and heterocyclic compounds are considered pivotal compounds in oils, contributing to the distinctive fragrance [12]. It has been reported that 2-methylpyrazine, 2,5-dimethylpyrazine, 2-ethyl-3-methylpyrazine, and 2,6-dimethylpyrazine could be produced by heating virgin rapeseed oil at 100 °C and contributed toasted, nutty, woody, and burnt profiles [13]. The aroma of cold-pressed and hot-pressed walnut oil was studied using GC-IMS and GC×GC-O-MS. It was concluded that the key aroma active compounds in cold-pressed walnut oil were identified as 1-octene-3-ol, cyclohexanol, and benzaldehyde. Conversely, the main aroma components in hot-pressed walnut oil were 3-methylbutyraldehyde, (E, E)-2, 4-nonadienal, and nonanal [14].

Gas chromatography–ion mobility spectrometry (GC-IMS), gas chromatography-olfactometry-mass spectrometry (GC-O-MS), and electronic nose (E-nose) are commonly employed in food aroma research for the detection of volatile compounds (VOCs) due to their low limits of detection, high selectivity, robust operational stability, rapid analysis capabilities, and cost-effectiveness [15,16]. GC includes both a mobile phase and a stationary phase. The components of the sample are separated in the chromatographic column based on their different forces in the two-phase system. IMS is a method capable of detecting volatile and semi-volatile compounds at parts per billion (ppb) [17]. These compounds are distinguished based on the mobility of ions in the electric field of neutral buffer gas under atmospheric pressure [18]. GC-IMS not only exploits the high separation capacity of GC but also capitalizes on the high sensitivity and rapid response properties of IMS. Olfactometry (O) employs human sense of smell as a detector to identify aroma contributions in food [19]. Combined with the advantages of GC-O and GC-MS, GC-O-MS enables rapid and accurate identification of VOCs while also allowing for the recording of substances’ aroma through olfactometer export [20,21].

In recent years, the combination of multiple analytical methods to identify the key aroma compounds in oils has been used in various studies of oil flavor substances. The results obtained from these different methods are cross-verified to enhance accuracy, scientific rigor, and reliability. For example, He et al. [12] used both GC-IMS and GC-MS to analyze the aroma substances generated during different microwave treatment times of camellia seed oil and found that the primary odors varied with different treatments. According to a report [21], analyses using GC-MS and GC-O revealed that aldehydes and pyrazines were the primary aroma active compounds in roasted walnut oil.

Yellow horn seed oil has garnered increasing attention due to its valuable nutrients and distinct aroma, yet there is still a gap in the study of its aroma substances. Therefore, this study aims to identify unknown VOCs in yellow horn seed oil using GC-IMS and GC-O-MS, followed by determining the key aroma compounds by evaluating their contribution. Additionally, the impact of the roasting temperature on the aroma of the yellow horn seed oil is also discussed. The results obtained from this study will provide an analytical, theoretical, and technical foundation for further research into the aroma profile of yellow horn seed oil.

## 2. Materials and Methods

### 2.1. Materials and Chemicals

Yellow horn seeds were provided in September 2022 by Shandong Woqi Agricultural Development Co. Ltd. (Shandong, China). The shell of the seed was removed and the kernel remained intact. The dried seed kernels with a moisture content of 10% were stored at 4 °C. Divinylbenzene/carboxen/polydimethylsiloxane-fiber (DVB/CAR/PDMS, 50/30 μm, 1 cm) headspace glass vials were purchased from Sigma-Aldrich (St. Louis, MO, USA).

The 2-octanol standard was obtained from Shanghai Maclin Biochemical Technology Co., Ltd. (Shanghai, China). n-ketones C_4_–C_9_ were purchased from Sinopharm Chemical Reagent Beijing Co., Ltd. (Beijing, China). n-alkanes C_7_–C_30_ were purchased from TCI (Beijing, China).

### 2.2. Sample Preparation

Following the method reported in [22,23] with slight modifications, the preparation of yellow horn seed oil involved weighing 150 g of peeled yellow horn kernels and roasting them at six different temperatures (120 °C, 130 °C, 140 °C, 150 °C, 160 °C, and 170 °C) in an oven for a duration of 25 min. Subsequently, the roasted yellow horn kernels were transferred to an automatic oil press for extraction. The obtained samples of yellow horn seed oil were centrifuged, and the supernatant was sealed in a glass vial at 4 °C and stored away from light. Three replicate experiments were performed for each sample preparation.

### 2.3. HS-GC-IMS Analysis

HS-GC-IMS analysis was conducted using the GC-IMS FlavourSpec^®^ Instrument (Gesellschaft für Analytische Sensorsysteme, Dortmund, Germany) equipped with an automatic sampler (CTC Analytics AG, Zwingen, Switzerland). GC is equipped with a DB-WAX metal capillary column (15 m × 0.53 mm, 1 μm). The detection method used in [12] was adapted with some modifications, whereby the initial flow rate was maintained at 2 mL/min for 5 min and increased to 100 mL/min for 20 min. Substances were eluted and separated in the column. Subsequently, the compounds were transferred and ionized in an ionization chamber with a ^3^H ionization source in the positive ion mode. The derived ions were driven into the drift tube (98 mm) at 45 °C with a linear voltage of 500 V/cm.

n-ketones C_4_–C_9_ were used as an external reference to calculate the retention index (RI) of VOCs detected under the same chromatographic conditions. VOCs were identified by comparing RI and drift time (DT) of standard compounds in the NIST 17 library and the GC-IMS database (G.A.S GmbH, Dortmund, Germany). LAV software (LAV, Dortmund, Germany) was used to quantitatively analyze the signal peak area of the samples detected using GC-IMS. The average peak areas were utilized to indicate the relative abundance of volatiles.

### 2.4. HS-SPME/GC-O-MS Analysis

#### 2.4.1. Extraction of Volatile Compounds

**Figure 1:**
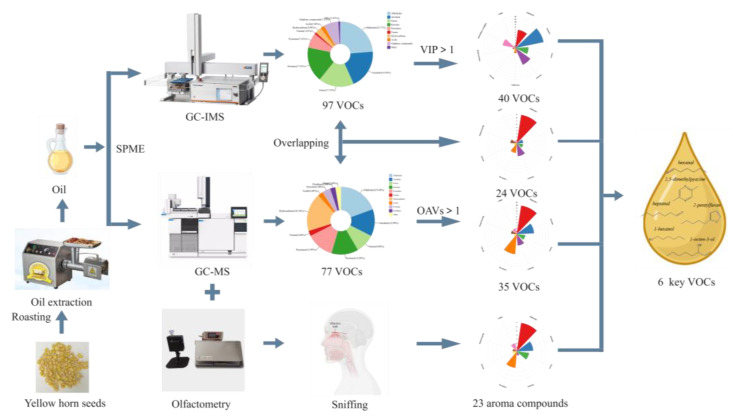
A scheme of the overall process to achieve the key aroma compounds.

Following the method reported in [14] with minor modifications, VOCs in yellow horn seed oil treated at different temperatures were isolated and analyzed using HS-SPME/GC-O-MS. The yellow horn seed oil (3.0 g) and 3 μL 2-octanol (25 mg/L methanol), which was an internal standard (IS), were added to a headspace vial. The SPME fibers (DVB/CAR/PDMS) were thoroughly exposed to the top of the headspace vial (20 mL) and extracted at 80 °C for 20 min. After extraction, the fibers with extracts were inserted into the injection port of the GC system in the splitless mode and desorbed at 250 °C for 10 min [24]—three times in parallel for each sample. As decripted in Figure 1.

#### 2.4.2. GC-O-MS Analysis of Volatile Compounds in Yellow Horn Seed Oil

A combination of 7890B-GC (Agilent Technologies, Inc., Santa Clara, CA, USA), olfactometer (Sniffer 9000, Brechbuhler, Schlieren, Switzerland), and 5977A-MS (Agilent Technologies, Inc., Santa Clara, CA, USA) was applied. VOCs were separated using a DB-5MS capillary column (30 m × 250 μm, 0.25 μm, Agilent Technologies Inc., Santa Clara, CA, USA). The chromatographic column temperature was set to 40 °C for 2 min, increased to 220 °C at a rate of 6 °C/min, then ramped up to 280 °C at a rate of 20 °C/min, and held for 10 min. The carrier gas was helium (purity ≥ 99.999%) at a flow rate of 3.0 mL/min. Ionization was carried out in the electron ionization mode at 70 eV, and the resulting mass spectrum obtained was in the 30–330 *m*/*z* range. The MS source temperature was kept at 230 °C. Three experienced panelists (two females and one male, aged between 22 and 25) were recruited to perform a sniff test on the olfactory output to identify aroma active compounds. Wet air (high-purity nitrogen and distilled water) was used for ventilation to improve the comfort of panelists. The terminal effluent from the capillary flowed into the MS and olfactometer with a split ratio of 1:1, respectively. Panelists were asked to record the perceived aroma, intensity, and time during sniffing.

MS characterization, odor description (O), and retention index (RI) were used for quantitative analysis [25]. MS characterization required that target compounds with a matching degree greater than 800 were screened from the NIST17 library based on the MS results. Odor description (O) was used to record the time and fragrance of smell on the sniffer port. The retention index of each compound was determined using the retention time of the n-alkanes C_7_-C_30_ by linear interpolation and compared to the RI of standard compounds in the NIST17 library [26]. The semi-quantization of the VOCs in the GC-O-MS sample was based on a linear relation between the peak area and the concentration of the IS and the concentration of the VOCs. The concentration was determined using the following equation.

(1) ρx=fx×AxA1×ρ1

$f_{x}$: correction factor of compound; $A_{x}$: peak area of unknown compound; $A_{1}$: peak area of internal standard; $\rho_{1}$: concentration of internal standard

### 2.5. Odor Active Values (OAVs)

The OAV is calculated based on the ratio of the concentration of the detected compound to its odor detection threshold in water [27], which is used as a visualization tool to determine the extent to which a compound contributes to food aroma [28]. VOCs with OAVs ≥ 1 are considered to contribute significantly to the aroma of yellow horn seed oil.

(2) OAVA=CATA

$C_{A}$: concentration of detected compound; $T_{A}$: odor threshold of corresponding compounds.

### 2.6. Statistical Analysis

The samples were collected in triplicate, and the resulting data were presented as mean ± standard deviation. Statistical analysis of the obtained data was performed using IBM SPSS 26 software (SPSS Inc., Chicago, IL, USA) and Origin Pro 2018 (OriginLab, Northampton, MA, USA). Data were subjected to one-way analysis of variance (ANOVA) using SPSS 26 software, and the multiple comparisons between the samples were performed via the Duncan test (*p* < 0.05). Partial least squares–discriminate analysis (PLS-DA) and orthogonal PLS-DA (OPLS-DA) were performed using SIMCA14.1 software (Umetrics, Umea, Sweden). The heat map was produced through the use of TBtools software (version v1.113).

## 3. Results

### 3.1. GC-IMS Analysis

#### 3.1.1. GC-IMS Topographic Plots and Fingerprints

**Figure 2:**
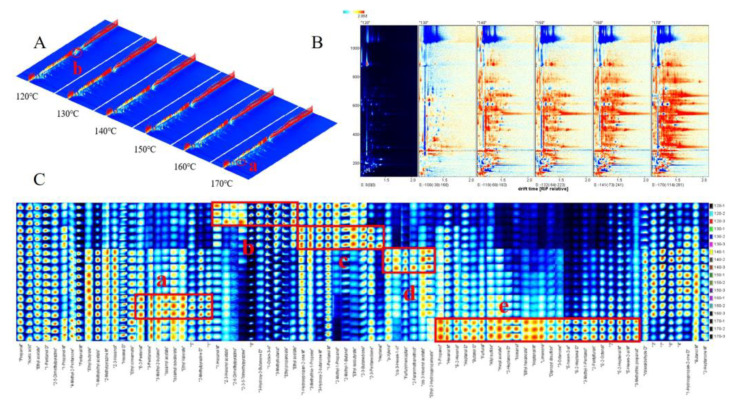
GC-IMS topographic plots and fingerprints of the yellow horn seed oil. (**A**) 3D topographic. (**B**) 2D topographic subtraction plots of the VOCs. (**C**) VOC fingerprint comparisons of the yellow horn seed oil.

GC-IMS utilizes ion mobility in the buffer to effectively separate and identify aroma compounds. Moreover, it enables rapid and accurate acquisition and visualization of IMS data for VOCs present in yellow horn seed oil. Figure 2A shows the 3D topographic of the VOCs in yellow horn seed oil roasted at different temperatures, with the red color indicating a higher content. Although the VOCs in yellow horn seed oil roasted at different temperatures exhibit similarities, there are slight variations in their signal intensity. As depicted in Figure 2A(a), an increase in roasting temperature leads to a rise in the majority of aroma substances. Additionally, certain constituents exhibit an inverse relationship with temperature, as depicted in Figure 2A(b). This finding is consistent with He et al.’s [12] study on a microwave treatment applied to rapeseed oil. The kinds and concentrations of VOCs were compared precisely in the range of 120–170 °C in a 2D topographic (Figure 2B). The reference spectrum at 120 °C was utilized, and the spectra of other temperatures were obtained via subtraction. The red area in the figure indicates a higher VOC content than the reference, while the blue indicates a lower concentration. As depicted in Figure 2B, the proportion of red compound area gradually increases with roasting temperature.

The VOC fingerprint of yellow horn seed oil was constructed to accurately represent the variations in VOC content resulting from different roasting temperatures (Figure 2C). Each row represents the signal peaks for different samples, while each column represents a distinct volatile compound. The brightness of the color is proportional to the VOC content, with higher concentrations resulting in a more intense red hue and lower concentrations, producing a brighter blue shade. Five areas (a, b, c, d, e) marked with wireframe exhibit elevated content of VOCs in the different samples of the yellow horn seed oil treated at 120–170 °C. The concentration of volatile components in a-region reached the highest in the sample of 160 °C, with nine VOCs present, including 2-pentenal (isomer), ethyl valerate and 2-methylpyrazine. The b-region exhibited the highest concentration of VOCs in the 120 °C sample, including 3-hydroxy-2-butanone ethyl acetate, 1-octen-3-ol and additional ten VOCs. Meanwhile, the c-zone was dominated by ten VOCs, such as 2,3-butanedione, 2,3-pentanedione and heptanal in the 130 °C sample. The d-region contained six VOCs, with the highest concentration observed in the 140 °C sample. These compounds included furfuryl mercaptan, 3-hexenyl acetate (isomer), 3-hexen-1-ol (isomer), etc. In contrast, the e-zone was dominated by 2-heptenal (isomer), 2-pentylfuran, and 2-octenal (isomer), among other VOCs. The 170 °C sample had the highest concentration of these aroma substances. The results above suggest that the volatile components in yellow horn seed oil samples roasted at different temperatures vary significantly (*p* < 0.05), indicating a significant impact of roasting temperature on the aroma profile of the oil.

#### 3.1.2. Volatile Compounds Analysis

GC-IMS is a novel separation and detection technology with the advantages of sensitivity, accuracy, and rapidity, which can be applied to the determination of aroma compounds in yellow horn seed oil [29]. A total of 178 chromatographic signals have been detected by using GC-IMS, leading to the identification of 97 aroma compounds in the GC-IMS database. These compounds consisted of 23 aldehydes, 19 alcohols, 17 ketones, two acids, 17 esters, seven sulfur-containing compounds, one furan heterocyclic compounds, three hydrocarbons, seven pyrazine heterocyclic compounds, and one other compound (Figure S1A). Meanwhile, some compounds exhibit multiple signals (monomer and dimer) due to the addition of ions and neutral molecules in GC-IMS [30]. These compounds have similar retention times but different drift times. Further details regarding the VOCs are presented in Table S1.

**Figure 3:**
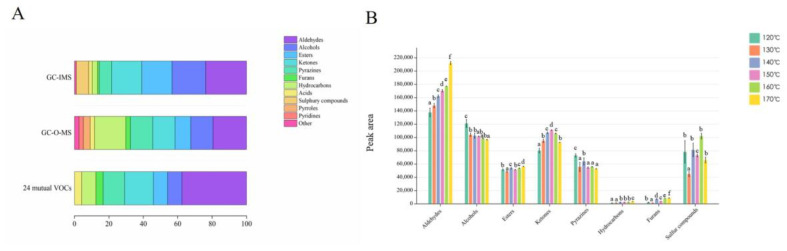
GC-IMS and GC-MS data diagram in the yellow horn seed oil at different temperatures. (**A**) Comparison of stacked bar for extraction of VOCs. (**B**) The peak area of each component in oil was measured via GC-IMS. Different lowercase letters (a–f) mean statistically significant differences (*p* < 0.05).

Aldehydes, which contribute to the roasted and fatty aroma, are key compounds found in plant raw materials such as peanuts and canola oil [31]. In yellow horn seed oil, aldehydes were the most abundant VOCs detected via GC-IMS, accounting for 23.71% of the total, followed by alcohols (19.59%) and ketones (17.53%) (Figure 3A). From Figure 3B, it can be seen that the content of aldehydes increased significantly with the increase in roasting temperature within a certain temperature range, such as 2-octenal (isomer), 2-heptenal (isomer), and nonanal (*p* < 0.05). The formation of these compounds is related to lipid oxidation. For instance, nonanal, octanal, and decanal are produced via the auto-oxidation of oleic acid (C1:18), generating 10-hydroperoxides and 11-hydroperoxides, and/or secondary formation of 8-hydroperoxide [32]. The findings suggest that the heating process of yellow horn seed leads to lipid oxidation, which leads to the formation of these aroma compounds. Alcohols could be produced via the oxidation of polyunsaturated fatty acids catalyzed by lipoxygenase [33,34]. Meanwhile, the amino acid derivation pathway was also an essential source of some alcohols [35]. For instance, phenylethyl alcohol could be synthesized from phenylalanine by the action of aromatic amino acid aminotransferase (AAAAT) [36]. Phenylethyl alcohol was found in low content. In contrast, the concentration of 1-octen-3-ol was higher, indicating that the alcohol was produced, presumably, via fat oxidation or aldehyde reduction in yellow horn seed oil roasted at different temperatures. This result was consistent with Xu et al.’s [14] opinion of the results in their study concerning walnut oil. Ketones are typically produced through the decomposition of amino acids and thermal oxidative degradation of polyunsaturated fatty acids [14]. As shown in Figure 3B, the ketone content tended to increase and then decrease with increasing temperature. The sample at 150 °C had the highest content, while that at 120 °C had the lowest. Seven sulfur-containing compounds, including dimethyl disulfide, dimethyl trisulfide, and diethyl disulfide, have been detected in yellow horn seed oil. These findings were consistent with previous reports, which identified dimethyl trisulfide as one of the important aroma compounds in their analysis of fragrant rapeseed oil [10].

#### 3.1.3. Multivariate Statistical Analysis

**Figure 4:**
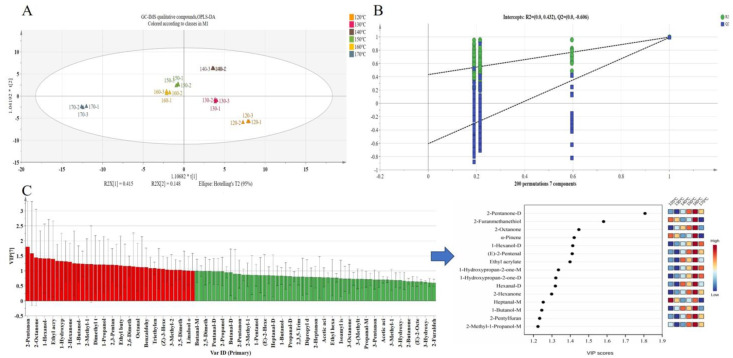
Analysis results of OPLS-DA model of GC-IMS data in the yellow horn seed oil roasted at different temperatures. (**A**) OPLS-DA model (R^2^Y = 0.915, Q^2^ = 0.908). (**B**) Cross-validation plot produced via 200 permutation tests (R^2^ = 0.432, Q^2^ = −0.606). (**C**) VIP values of VOCs (red represents 40 key compounds with VIP > 1).

The OPLS-DA model is capable of effectively highlighting inter-group sample differences, thereby enabling the classification information to be focused primarily on a single principal component, which enhances the analytical capability and validity of the model. When model parameters R^2^Y and Q^2^ are between 0.5 and 1, the model is considered optimal [37]. In the present study, the OPLS-DA model was developed using GC-IMS data from yellow horn seed oil. The parameters of the OPLS-DA model, R^2^Y = 0.915 and Q^2^ = 0.908, illustrated that the results had a good interpretation rate and predictability (Figure 4A). Additionally, 200 iterations of the permutation test (permutation test R^2^ = 0.432, Q^2^ = −0.606) fitted well without over-fitting, achieving rapid discrimination of the yellow horn seed oil at different temperatures (Figure 4B). The overall distribution of the six samples was scattered. The 120 °C and 130 °C samples were distributed in the fourth quadrant. Both the 150 °C and 160 °C samples were distributed close to each other in the second quadrant, indicating that their VOCs were relatively similar. The 140 °C samples were distributed in the first quadrant alone, and the 170 °C samples were distributed in the third quadrant alone, indicating that their VOCs differed greatly in similarity.

Variable importance in projection (VIP) describes the overall contribution of each variable to the model. Therefore, VIP served as a means to evaluate the strength and explanatory power of individual variables with respect to classification and discrimination [38]. In this study, a screening criterion of VIP > 1 was employed to identify compounds that significantly contributed to the sample classification results. As shown in Table 1, there were 40 key VOCs with VIP > 1. VOCs with VIP > 1 were highlighted in red in Figure 4C, including seven aldehydes, 11 alcohols, two pyrazine heterocyclic compounds, five esters, seven ketones, five sulfur-containing compounds, and one other compound. The shift in the content of these VOCs resulted in a change in the classification of the yellow horn seed oil produced at 120–170 °C.

### 3.2. GC-O-MS Analysis

#### 3.2.1. Volatile Compounds Analysis

By integrating instrumental and sensory analysis, GC-O-MS is now capable of a more comprehensive analysis of the compounds. Meanwhile, the human nose is also used as an aroma detector, which was combined with sensory analysis to realize the transformation from chemical composition analysis to aroma analysis [39]. A total of 77 VOCs were detected via GC-O-MS, consisting of fifteen aldehydes, fourteen hydrocarbons, ten alcohols, ten ketones, two acids, seven esters, ten pyrazine heterocyclic compounds, two furan heterocyclic compounds, and seven other compounds (Figure S1C). Aldehydes were the most diverse, accounting for 19.48% of the total compounds. This was followed by hydrocarbons (18.18%), pyrazines (12.99%), ketones (12.99%), and alcohols (12.99%). As illustrated in Figure S1D, the difference in the VOCs determined in the oil via GC-O-MS and GC-IMS can be related to the affinity of the VOCs to the rest phase of these two GC columns.

Based on the odor recordings, the time of the occurrence of the aromas was combined with the peak time of the VOCs, and the olfactory outcomes were obtained by matching the sniffed compounds. In conjunction with sensory evaluation results, a total of 23 aroma substances were identified, including seven aldehydes, four alcohols, three esters, one ketone, four pyrazine heterocyclic compounds, one furan heterocyclic compound, one hydrocarbon, and two other compounds (Table 2). The main aroma characteristics included nutty, oily, green, grassy, earthy, and mushroom profiles. Among aroma compounds, aldehydes [40] contribute to a fresh and fatty profile, while pyrazine and furan impart a nutty aroma [41]. In addition, grassy and fruity profiles were predominantly provided by alcohols and esters.

The overall aroma of a food product is influenced by its aroma active compounds, which are determined by their concentration and odor threshold. OAVs can be used to identify the key contributors to the aroma profile of food products, making it a valuable screening tool in aroma studies [42]. OAVs ≥ 1 indicate that the compound contributes significantly to the overall aroma profile of food. The higher the OAV, the more prominent its contribution to food aroma [43]. The concentration of VOCs was determined via semi-quantization. The OAV of each compound was calculated as the ratio between its concentration and the corresponding odor threshold, and the results were summarized in Table S2. OAVs ≥ 1 were used as the screening criteria for the aroma distribution of the yellow horn seed oil in this study, and 35 compounds were screened (Table 2). Possible synergistic or additional complex interactive effects may not be explained accurately since only considering OAVs > 1. However, OAVs > 1 can predict the overall aroma characteristics of roasting yellow horn seed oil at different temperatures [44].

**Figure 5:**
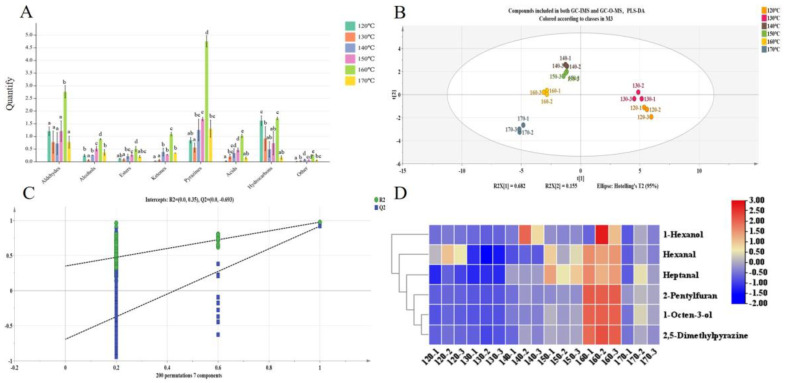
PLS-DA analysis results of GC-O-MS and GC-IMS in the yellow horn seed oil roasted at different temperatures and Heat map of 6 key aroma compounds. (**A**)The peak area of each component in oil was measured via GC-IMS. (**B**) PLS-DA model (R^2^Y = 0.979, Q^2^ = 0.904). (**C**) Cross-validation plot by 200 permutation tests (R^2^ = 0.35, Q^2^ = −0.693). (**D**) Heat map of 6 key aroma compounds. Different lowercase letters (a–e) mean statistically significant differences (*p* < 0.05).

Aldehydes: The yellow horn seed oil exhibited the highest concentration of volatile aldehydes, as detected via GC-O-MS at 160 °C, with a concentration of 2.7649 mg/kg (Figure 5A). A total of 10 aldehydes with OAVs ≥ 1 were identified, including isobutyraldehyde (fresh, aldehydic, floral, and green), 3-methylbutanal (ethereal, chocolate, peach, and fatty), pentanal (fermented, bready, fruity, nutty, and berry), hexanal (fresh, green, fatty, grass, and fruity), heptanal (fresh, green, fatty, fruity, and sweaty), octanal (green, herbal, fresh, and fatty), (E)-2-octenal (fresh, cucumber, fatty, and green), nonanal (fresh, orange, peel, and fatty), (E)-2,4-decadienal (fried), (E,E)-2,4-decadienal (earthy, fried, and oily), providing fatty, nutty, grass, fruity and green for the roasted yellow horn seed oil. Among the aldehydes, the OAV of isobutyraldehyde was the highest in the 160 °C sample, up to 757.60. Previous studies have indicated that isobutyraldehyde was mainly produced via the Strecker degradation of isoleucine, leucine, and valine and provided maltiness to the aroma distribution of the oil [45,46]. Nonanal, octanal and hexanal have been reported to provide grass and fatty in rapeseed oil [45]. Benzeneacetaldehyde, which has a sweet and rose-like aroma, is a vital product of the shikimate pathway [47]. According to the above results, it is believed that the detected aldehydes contribute significantly to the aroma profile of the oil.

Alcohols: Alcohols can be generated through either lipid oxidation or aldehyde reduction, as previously reported [48]. The highest alcohol content of 1.0126 mg/kg was observed in the sample heated at 160 °C. The VOCs with OAVs ≥ 1 included 1-hexanol (ethereal, oil, fruity, sweet, green) and 1-octene-3-ol (mushroom, earthy). 1-hexanol (1.79–15.81%) and 1-octene-3-ol (2.09–4.95%) were also found to be important alcohol compounds in cold-pressed peanut oil [40]. Aroma compounds were determined by comparing the combined time of olfactometry-based aroma detection with the peak retention times of the GC-O-MS-detected compounds. Distinct scents of grass, mushrooms, and earthy notes are perceptible during the peak production period of these aroma compounds.

Esters: Studies have reported that most esters were associated with fruity and sweet aromas [49]. Volatile esters were formed via the esterification of various alcohols and carboxylic acids and/or esterification, which was catalyzed by using alcohol acyltransferases [50]. The content of ester components showed the maximum value (0.51 mg/kg) in the 160 °C sample and the minimum value (0.099 mg/kg) in the 130 °C sample. Among the yellow horn seed oil, three ester compounds with OVAs ≥ 1 were identified, including butyl acetate (ethereal, solvent, fruity, and banana), ethyl 2-methylbutyrate (fruity) and isoamyl acetate (sweet, fruity, banana, and solvent). Combined with olfactory analysis, it was determined that fruity aromas are distinctly detectable during their peak period.

Pyrazines: With increasing roasting temperature, the content of pyrazine compounds in yellow horn seed oil VOCs significantly changed (*p* < 0.05), reaching a peak (4.76 mg/kg) in the sample roasted at 160 °C. The VOCs with OAVs ≥ 1 contributed significantly to food aroma distribution. Seven Pyrazine compounds with OAVs ≥ 1 were screened from the yellow horn seed oil, including 2,5-dimethylpyrazine (roasted, nutty, popcorn), 2-mthyl-6-methylpyrazine (roasted, baked potato), 2,3,5-trimethylpyrazine (roasted, nutty), 3-ethyl-2,5-dimethylpyrazine (roasted, nutty), 2-ethyl-3,5-dimethylpyrazine (nutty, roasted, sweet), 2,6-diethylpyrazine (roasted, nutty, sweet), and 2,3-diethyl-5-methylpyrazine (raw peanut, potato). 2, 6-diethylpyrazine, an important product of the Maillard reaction [51], has been identified as a key aroma compound in sesame oil [11] and virgin rapeseed oil [52], providing nutty and toasty profiles. The results suggest that the presence of these aroma compounds contributes to the development of a nuanced roasted and nutty aroma in yellow horn seed oil.

Ketones: Ketones are typically generated through amino acid decomposition and the thermal oxidative degradation of polyunsaturated fatty acids [14]. Ketones with carbon numbers below seven were VOCs derived from lipids, contribute to fatty, herbal, flowery, and sweet aromas in samples [53]. The contents of ketones were the highest in the 160 °C sample (1.0949 mg/kg) in the aroma assay of the yellow horn seed oil roasted at different temperatures. There were three aroma substances with OAVs ≥ 1, including acetoin (sweet, buttery, and creamy), 4-hydroxy-2, 5-dimethyl-3 (2H) -furanone (sweet, soap, and bread), and 2-decanone (soap). Acetoin, as a short-chain ketone, could be derived from pyruvate conversion and citrate metabolism and/or conversion of diacetyl by diacetyl reductase enzyme [54]. Meanwhile, He et al. [12] confirmed that acetoin was a key aroma compound, providing a buttery odor to camellia seed oil. 4-hydroxy-2, 5-dimethyl-3 (2H)-furanone was a product of the Maillard reaction [55], providing fruitiness and sweetness profiles to yellow horn seed oil. 2-decanone possesses a soap aroma in yellow horn seed oil, mainly formed from the degradation of linoleic acid [56].

Hydrocarbons: Hydrocarbons were mainly derived from the decomposition of fatty acids alkoxy groups [14]. The total hydrocarbon content was the highest in the 160 °C sample, with a content of 1.7149 mg/kg in yellow horn seed oil. The OAVs of the unsaturated and saturated hydrocarbons in yellow horn seed oil are mostly low due to their high odor threshold, which limits their contribution to food aroma. Only the OAV (3.64) of styrene (sweet, balsamic, floral, and plastic) exceeds 1 in the 120 °C sample, imparting an herbal and citrus aroma to the oil.

#### 3.2.2. Olfactometry Results

According to the combination of olfactory time and gas chromatography–retention time analysis, 31 compounds were screened out, including five alcohols (16.13%), three esters (9.68%), eight aldehydes (25.81%), two ketones (6.45%), six pyrazines heterocyclic compounds (19.35%), one furan heterocyclic compounds (3.23%), two hydrocarbons (6.45%), and four others (12.90%) (Table S3). Among them, 23 aroma compounds exhibited an OAV greater than 1 (Table 2). These compounds, which possess distinct aromas, are considered as significantly contributing to the overall aroma profile of yellow horn seed oil. Strong oily, fatty, and nutty odors are provided by the aroma compounds, such as 2-octenal, nonanal, and 2,4-decadienal in yellow horn seed oil. The grassy, green, and fruity aromas are mainly provided by aroma compounds, such as hexanol, 1-octen-3-ol, hexanal, 2-pentylfuran, and ethyl 2-methylbutyrate. Studies have reported that 2,4-decadienal provided a pleasant oily fragrance in fragrant rapeseed oil [57]. Similarly, nonanal is an oxidation product of oleic acid and contributes an oily aroma to food, as reported in [32,58]. (E)-2-octenal, which is a product of the oxidation of linoleic acid, was detected in nut oil [59]. (E)-2-octenal presented elevated content and low odor threshold, causing the nut oil to have its distinctive fatty aroma. 1-octen-3-ol and 2-pentylfuran were also detected in walnut oil and provided a green aroma in relation to walnut oil [60]. In this study, the synergistic effect of these aroma compounds contributed to the development of a robust aroma profile in hot-pressed yellow horn seed oil.

### 3.3. Combined Analysis of HS-SPME× GC-O-MS and HS-GC-IMS

#### 3.3.1. Results of GC-O-MS and GC-IMS Combined Analysis

Through the analysis of GC-O-MS and GC-IMS data, it was found that 24 compounds were detected through the use of both methods (Figure S2A), including nine aldehydes (37.50%), four ketones (16.67%), three pyrazine heterocyclic compounds (12.50%), two esters (8.33%), two alcohols (8.33%), two hydrocarbons (8.33%), one furan heterocyclic compound (4.17%), and one acid (4.17%). As depicted in Figure S2B, the six groups of samples exhibited discernible discrepancies. Notably, the abundances of p-Xylene and Isoamyl acetate were higher in the 120 °C sample than those in other groups. However, the overall abundance was highest in the 160 °C samples. These results suggest that treatment at 160 °C is optimal for achieving maximum volatile compound abundance.

The PLS-DA model was established using data from 24 compounds detected via GC-IMS and GC-O-MS, as illustrated in Figure 5B. The separation of six samples is clearly observed in the PLS-DA model. The model parameters were R^2^Y = 0.979 and Q^2^ = 0.904, which meant the PLS-DA model was reliable. Meanwhile, as shown in Figure 5C, the results of 200 permutation tests indicated a good model fit with no evidence of overfitting (R^2^ = 0.35, Q^2^ = −0.693). Specifically, the 120 °C sample and 130 °C sample were distributed in the fourth quadrant, indicating their VOCs were relatively similar. The 140 °C, 150 °C, and 160 °C samples were distributed in the third quadrant, suggesting that their VOCs were relatively similar. In contrast, the 170 °C sample was distributed in the third quadrant alone, which indicates that the content of the 170 °C sample was significantly different from other samples. The six samples could be divided into three categories: the 120 °C and 130 °C samples belong to one group, while the 140 °C, 150 °C, and 160 °C samples from another group. The remaining sample is categorized separately at a temperature of 170 °C. The results were consistent with the OPLS-DA model established via GC-IMS.

#### 3.3.2. Determination of the Key Aroma Compounds in Yellow Horn Seed Oil

Forty kinds of VOCs with VIP > 1 in GC-IMS and 23 kinds of main aroma compounds with combined OAVs ≥ 1 and sniffing in GC-O-MS were selected as screening criteria. Six VOCs were screened, including hexanal, 2,5-dimethylpyazine, heptanal, 2-pentylfuran, 1-hexanol and 1-octen-3-ol. They were considered to be the key aroma compounds in yellow horn seed oil. Hexanal in flaxseed oil contributes fatty, oily, sweet, nutty, and green odors [61]. It was reported that 2,5-dimethylpyrazine was a key aroma compound in oils such as canola oil [62], nut oil [59], and olive oil [13] and provided roasted and nutty aromas. In a roasted peanut oil study conducted by Yang et al. [63], it has been confirmed that heptanol is the product of fatty-acid oxidation and one of the important aroma compounds, providing fatty and oily aromas for roasted peanut oil. 2-pentylfuran is formed via the cyclization of peroxidation free radicals in the oxidation process of linoleic acid [64], which was considered as an important index of lipid oxidation. 2-pentylfuran can impart fruity and green aromas to the yellow horn seed oil. Through thermal oxidation studies on different varieties of olive oil, Kiralan et al. [65] reported that 2-pentylfuran was found in thyme-flavored olive oil during thermal oxidation. Yang et al. [66] reported that 1-hexanol provided a typical fresh odor for raw flaxseed powders, giving green-like, fresh-like, mint-like aromas via the aroma description of a microwave treatment of flaxseed meal. It was reported that linoleic acid (C2:10)-derived 10-L(S)-hydroperoxy-cis-9 and trans-11-octadecadieuoic acid via enzymatic oxidation, which then converted to 1-octen-3-ol [32]. Meanwhile, 1-octen-3-ol mainly contributed to mushroom and sweet odors to food. As shown in Figure 5D, the abundances of hexanal and heptanal in the 150 °C and 160 °C samples were significantly different from the other groups (*p* < 0.05). Moreover, 2-pentylfuran, 1-octen-3-ol, and 2,5-dimethylpyrazine in the 160 °C sample were significantly more abundant than in the other groups. To sum up, these six VOCs were important aroma substances that provided key aromas to yellow horn seed oil.

## 4. Conclusions

In this study, the aroma composition of hot-pressed yellow horn seed oil was assayed by using the seed of yellow horn roasted at different temperatures. VOCs were identified using HS-GC-IMS and HS-SPME/GC-O-MS. GC-IMS allowed for the determination of 97 VOCs, while GC-O-MS allowed for the determination of 77 VOCs, and both methods allowed for the identification of 24 common VOCs. In addition, the OAV values were calculated, and 35 VOCs were screened by OAVs ≥ 1 as the standard. Subsequently, twenty-three aroma compounds were confirmed through the use of olfactory probe recordings.

2,5-dimethylpyrazine, hexanal, heptanal, 2-pentylfuran, hexanol and 1-octen-3-ol were determined as the key aroma compounds in yellow horn seed oil by VOCs with VIP > 1 in GC-IMS and aroma compounds with combined OAVs ≥ 1 and olfactometry in GC-O-MS. Combining the result of OPLS-DA and PLS-DA, it was concluded that the aroma compounds of the samples were similar at 120 °C and 130 °C, while those of the samples were similar at 140 °C, 150 °C and 160 °C. According to the results of GC-O-MS, the VOC content of the sample at 160 °C is higher. The results of the present study supplemented the understanding of the composition and aroma of VOCs in yellow horn seed oil.

## Figures and Tables

**Table 1:** Volatile compounds identified of VIP > 1 via GC-IMS in yellow horn seed oil.

No.	Compounds	Odor Description	CAS	Formula	MW	RI	Rt [sec]	Dt [RIP rel]	Peak Intensities						VIP
									120 °C	130 °C	140 °C	150 °C	160 °C	170 °C	
1	Allyl methyl sulfide	Sulfurous	10152-76-8	C_4_H_8_S	88.2	956.4	212.524	1.0444	8057.49 ± 57.43 ^b^	9167.78 ± 246.58 ^c^	8105.53 ± 580.62 ^b^	6730.20 ± 90.04 ^a^	5993.88 ± 137.32 ^a^	6654.80 ± 140.59 ^a^	1.19
2	Dimethyl disulfide	Moldy, onion-like, putrid, unpleasant, cabbage-like	624-92-0	C_2_H_6_S_2_	94.2	1095.1	308.773	1.1483	52,366.17 ± 3599.12 ^b^	23,361.85 ± 1372.65 ^a^	47,382.14 ± 5800.59 ^b^	46,471.18 ± 543.85 ^b^	71,141.45 ± 3969.37 ^c^	39,825.86 ± 3902.28 ^b^	1.22
3	Dimethyl trisulfide	Sulphury, cabbage	3658-80-8	C_2_H_6_S_3_	126.3	1412.7	961.969	1.2937	540.84 ± 31.16 ^a^	497.46 ± 22.96 ^a^	760.40 ± 50.73 ^c^	618.64 ± 14.33 ^b^	638.54 ± 43.64 ^b^	882.27 ± 19.98 ^d^	1.21
4	Diethyl disulfide	Onion, moldy, sulfur	110-81-6	C_4_H_10_S_2_	122.2	1186.5	421.014	1.1476	10,791.32 ± 952.54 ^cd^	4143.10 ± 316.85 ^a^	9578.81 ± 909.06 ^bc^	8562.63 ± 402.53 ^bc^	12,593.85 ± 454.33 ^d^	7971.58 ± 694.98 ^b^	1.15
5	2-Furfurylthiol	Coffee-like	98-02-2	C_5_H_6_OS	114.2	1399.6	917.063	1.1071	5801.82 ± 65.94 ^a^	6862.4 ± 134.06 ^b^	14,499 ± 1525.19 ^f^	9227.04 ± 77.74 ^d^	10,126.39 ± 174.88 ^e^	8020.17 ± 153.08 ^c^	1.58
6	Hexanal-M	Fresh, green, fatty, aldehydic, grass, leafy, fruity, sweaty	66-25-1	C_6_H_12_O	100.2	1096	309.662	1.5677	7780.20 ± 1173.47 ^a^	9160.00 ± 228.77 ^b^	10,374.69 ± 2078.82 ^b^	10,037.92 ± 274.70 ^ab^	8993.22 ± 430.72 ^ab^	13,609.09 ± 407.11 ^ab^	1.04
7	Hexanal-D	Fresh, green, fatty, aldehydic, grass, leafy, fruity, sweaty	66-25-1	C_6_H_12_O	100.2	1094.6	308.338	1.2575	11,718.59 ± 1742.32 ^a^	13,205.83 ± 223.23 ^ab^	13,222.23 ± 788.05 ^b^	12,805.39 ± 134.00 ^b^	12,532.19 ± 152.99 ^ab^	12,756.45 ± 136.71 ^c^	1.32
8	2-Pentenal (isomer)	Pungent, green, fruity, apple, orange, tomato	1576-87-0	C_5_H_8_O	84.1	1143.8	362.171	1.1088	6509.70 ± 1184.66 ^a^	10,950.67 ± 883.95 ^c^	6262.04 ± 311.96 ^a^	6272.97 ± 491.38 ^a^	6794.17 ± 455.91 ^a^	8605.5 ± 479.22 ^b^	1.41
9	Heptanal-M	Fresh, aldehydic, fatty, green, herbal, wine-lee, ozone	111-71-7	C_7_H_14_O	114.2	1195.1	434.412	1.3307	4318.85 ± 670.22 ^a^	7012.96 ± 1232.49 ^a^	4257.53 ± 460.87 ^a^	4610.66 ± 255.91 ^b^	4856.44 ± 198.56 ^c^	6485.02 ± 371.22 ^d^	1.25
10	Benzaldehyde	Strong, sharp, sweet, bitter, almond, cherry	100-52-7	C_7_H_6_O	106.1	1469.1	1182.187	1.4699	1458.55 ± 416.72 ^bcd^	988.42 ± 194.81 ^a^	1227.33 ± 92.57 ^abc^	1059.22 ± 99 ^ab^	1519.44 ± 95.97 ^d^	1792.55 ± 209.82 ^d^	1.12
11	2,4-Heptadienal (isomer)	Fatty, green, oily, aldehydic, vegetable, cake, cinnamon	4313-03-5	C_7_H_10_O	110.2	1452.4	1112.091	1.2001	3859.48 ± 455.45 ^c^	1762.31 ± 443.30 ^ab^	1484.30 ± 338.59 ^ab^	1720.04 ± 30.69 ^ab^	2233.30 ± 26.19 ^b^	3426.50 ± 157.52 ^c^	1.21
12	Octanal	Aldehydic, waxy, citrus, orange, peel, green, herbal, fresh, fatty	124-13-0	C_8_H_16_O	128.2	1313.9	670.399	1.407	572.35 ± 60.76 ^b^	495.32 ± 58.00 ^a^	1166.22 ± 24.64 ^e^	816.25 ± 37.78 ^c^	1045.30 ± 6.54 ^d^	1165.11 ± 24.56 ^e^	1.13
13	1-Octen-3-ol	Mushroom, earthy	3391-86-4	C_8_H_16_O	128.2	1467.6	1175.894	1.1584	40,475.67 ± 3853.92 ^c^	19,298.01 ± 3456.23 ^b^	20,171.27 ± 4552.03 ^b^	13,541.02 ± 852.27 ^a^	14,032.07 ± 158.17 ^a^	11,865.48 ± 350.39 ^a^	1.09
14	Linalool oxide	Musty, camphor, fenchyl, alcohol	60047-17-8	C_10_H_18_O_2_	172.3	1441.1	1069.247	3.2598	9670.70 ± 3053.56 ^b^	3190.54 ± 1158.91 ^a^	4354.89 ± 332.61 ^a^	3202.59 ± 56.06 ^a^	3747.35 ± 95.80 ^a^	1785.63 ± 101.36 ^a^	1.00
15	1-Propanol-M	Fermented	71-23-8	C_3_H_8_O	60.1	1046.3	267.072	1.1112	4436.79 ± 1190.25 ^a^	6565.21 ± 172.18 ^cd^	4900.08 ± 461.96 ^ab^	5768.09 ± 73.02 ^bc^	4305.7 ± 216.30 ^a^	6820.04 ± 221.51 ^d^	1.21
16	1-Butanol-M	Fusel oil, sweet, balsam, whiskey	71-36-3	C_4_H_10_O	74.1	1154	375.02	1.185	3744.79 ± 598.76 ^a^	4548.49 ± 70.81 ^b^	4686.03 ± 40.04 ^bc^	5376.44 ± 93.79 ^d^	4730.05 ± 141.61 ^bc^	5125.45 ± 171.36 ^cd^	1.25
17	2-Propanol-D	Alcohol, musty, woody	67-63-0	C_3_H_8_O	60.1	951.8	210.309	1.0803	3130.59 ± 296.88 ^a^	2748.43 ± 184.86 ^ab^	1158.02 ± 332.61 ^b^	1562.82 ± 68.13 ^ab^	1561.88 ± 73.44 ^b^	2819.65 ± 104.31 ^c^	1.01
18	2-Methyl-1-Propanol-M	Cortex	78-83-1	C_4_H_10_O	74.1	1104.2	317.826	1.1724	1732.68 ± 65.95 ^d^	1659.07 ± 82.18 ^d^	591.18 ± 140.40 ^b^	464.49 ± 32.80 ^ab^	1022.75 ± 133.15 ^c^	383.79 ± 30.18 ^a^	1.22
19	3-Methyl-2-Butanol	Fruity	598-75-4	C_5_H_12_O	88.1	1117.7	331.949	1.2297	1242.74 ± 51.75 ^b^	1040.34 ± 77.97 ^a^	1261.27 ± 165.58 ^b^	1619.70 ± 40.50 ^c^	2594.03 ± 50.09 ^d^	2692.83 ± 123.78 ^d^	1.03
20	1-Pentanol-D	Sweet, fruity	71-41-0	C_5_H_12_O	88.1	1258	546.611	1.5096	6814.24 ± 565.97 ^cd^	7150.80 ± 147.04 ^d^	6900.36 ± 179.89 ^cd^	8311.13 ± 18.77 ^c^	8208.22 ± 106.53 ^b^	9652.34 ± 183.83 ^a^	1.07
21	2-Methyl-1-Butanol-D	Alcohol, cocoa	137-32-6	C_5_H_12_O	88.1	1195	434.286	1.4677	3365.44 ± 465.36 ^cd^	3718.91 ± 159.65 ^d^	3108.47 ± 262.32 ^abc^	3256.73 ± 82.89 ^bc^	2698.04 ± 27.94 ^a^	2879.26 ± 99.53 ^ab^	1.00
22	1-Hexanol-M	Ethereal, fusel oil, fruity, alcoholic, sweet, green	111-27-3	C_6_H_14_O	102.2	1333.4	719.934	1.3277	3652.99 ± 251.84 ^e^	2928.45 ± 150.88 ^d^	2126.46 ± 137.32 ^ab^	2337.14 ± 24.83 ^bc^	1968.21 ± 27.93 ^a^	2486.17 ± 58.16 ^c^	1.03
23	1-Hexanol-D	Ethereal, fusel oil, fruity, alcoholic, sweet, green	111-27-3	C_6_H_14_O	102.2	1333.4	719.934	1.6422	3862.64 ± 426.21 ^a^	9007.31 ± 1099.47 ^b^	8640.73 ± 633.52 ^b^	11,297.05 ± 243.94 ^c^	8925.33 ± 179.25 ^b^	3793.13 ± 137.59 ^a^	1.41
24	2,5-Dimethylpyrazine-D	Roasted, nutty, popcorn	123-32-0	C_6_H_8_N_2_	108.1	1309.4	659.557	1.4993	758.27 ± 31.44 ^b^	605.49 ± 17.87 ^a^	966.26 ± 22.28 ^b^	1200.64 ± 25.78 ^c^	1293.21 ± 47.29 ^d^	1177.07 ± 14.31 ^e^	1.03
25	2,6-Dimethylpyrazine	Ethereal, cocoa, nutty, roasted, roasted, meaty, beefy, brown, coffee, buttermilk	108-50-9	C_6_H_8_N_2_	108.1	1333.1	719.115	1.5327	397.11 ± 10.40 ^b^	204.19 ± 14.61 ^a^	160.77 ± 34.96 ^a^	187.28 ± 8.70 ^a^	179.17 ± 37.07 ^a^	187.71 ± 26.50 ^a^	1.16
26	Triethylenediamine	−	280-57-9	C_6_H_12_N_2_	112.2	1501.8	1332.119	1.1697	6224.55 ± 1178.89 ^c^	2280.61 ± 535.37 ^ab^	2968.33 ± 1143.49 ^b^	1486.64 ± 121.80 ^a^	1365.63 ± 17.25 ^a^	1285.78 ± 107.58 ^a^	1.09
27	Ethyl butyrate	−	105-54-4	C_6_H_12_O_2_	116.2	1058.1	276.257	1.2052	2012.62 ± 106.02 ^a^	1829.35 ± 26.33 ^a^	2960.39 ± 376.09 ^c^	3076.36 ± 58.21 ^c^	2673.53 ± 166.98 ^b^	2848.26 ± 97.28 ^bc^	1.18
28	Isoamyl acetate	Sweet, fruity, banana, solvent	123-92-2	C_7_H_14_O_2_	130.2	1103.5	317.1	1.2968	396.85 ± 38.83 ^a^	359.45 ± 25.95 ^a^	597.33 ± 57.43 ^b^	665.35 ± 29.26 ^c^	1064.00 ± 20.07 ^e^	838.20 ± 31.48 ^d^	1.12
29	3-Hexen-1-ol acetate (isomer)	fresh, fruity	3681-71-8	C_8_H_14_O_2_	142.2	1300	637.406	1.2983	2279.16 ± 78.54 ^b^	1889.73 ± 68.43 ^a^	3053.78 ± 267.57 ^d^	2778.15 ± 84.63 ^c^	2826.48 ± 58.66 ^c^	2143.88 ± 59.70 ^b^	1.07
30	Ethyl lactate	Whey, creamy	97-64-3	C_5_H_10_O_3_	118.1	1313.4	669.166	1.5483	562.53 ± 102.08 ^a^	554.5 ± 22.12 ^a^	1687.33 ± 11.23 ^e^	1144.36 ± 43.15 ^c^	1277.58 ± 64.71 ^d^	783.55 ± 58.83 ^b^	1.20
31	Ethyl acrylate	−	140-88-5	C_5_H_8_O_2_	100.1	1003.1	237.762	1.1292	522.31 ± 15.9 ^d^	665.01 ± 46.32 ^e^	431.57 ± 27.36 ^ab^	419.40 ± 12.80 ^a^	469.79 ± 3.59 ^bc^	487.12 ± 12.09 ^cd^	1.40
32	2-Pentanone-D	Sweet, fruity, ethereal, wine, banana, woody	107-87-9	C_5_H_10_O	86.1	1029.4	254.809	1.3681	408.64 ± 87.77 ^bc^	346.98 ± 27.84 ^ab^	369.95 ± 13.41 ^bc^	441.01 ± 57.32 ^c^	275.33 ± 16.95 ^a^	551.34 ± 22.48 ^d^	1.81
33	4-Methyl-2-Pentanone	−	108-10-1	C_6_H_12_O	100.2	1024.6	251.512	1.1807	2700.01 ± 329.88 ^a^	3277.40 ± 69.74 ^bc^	3529.54 ± 108.52 ^c^	3502.25 ± 31.06 ^c^	3046.05 ± 74.26 ^b^	3889.29 ± 72.68 ^d^	1.17
34	2,3-Pentanedione	Caramel, buttery, sweet	600-14-6	C_5_H_8_O_2_	100.1	1074.4	289.875	1.2225	4558.31 ± 2449.92 ^a^	13,092.33 ± 308.38 ^d^	10,324.22 ± 1396.57 ^c^	11,519.26 ± 235.03 ^cd^	8022.99 ± 433.80 ^b^	11,561.28 ± 335.72 ^cd^	1.20
35	2-Hexanone	Soapy, banana	591-78-6	C_6_H_12_O	100.2	1071.2	287.036	1.1908	656.73 ± 128.08 ^a^	647.08 ± 76.99 ^a^	1114.34 ± 277.36 ^b^	1527.29 ± 38.34 ^c^	1425.84 ± 144.80 ^c^	1041.54 ± 26.66 ^b^	1.30
36	1-Hydroxypropan-2-one-M	Berry, sweet	116-09-6	C_3_H_6_O_2_	74.1	1301.8	641.394	1.0434	13,614.72 ± 791.77 ^a^	18,528.55 ± 693.32 ^d^	16,564.74 ± 415.40 ^b^	17,575.49 ± 100.62 ^c^	16,455.47 ± 62.10 ^b^	13,047.75 ± 275.54 ^a^	1.34
37	1-Hydroxypropan-2-one-D	Berry, sweet	116-09-6	C_3_H_6_O_2_	74.1	1287.8	609.588	1.2362	16,409.52 ± 1643.48 ^b^	16,502.61 ± 467.65 ^b^	15,641.14 ± 1495.48 ^b^	18,870.41 ± 302.10 ^c^	16,271.60 ± 197.92 ^b^	7496.50 ± 368.51 ^a^	1.32
38	2-Octanone	Herbal	111-13-7	C_8_H_16_O	128.2	1314.2	671.249	1.3332	248.09 ± 23.85 ^ab^	297.45 ± 9.64 ^b^	822.91 ± 100.36 ^e^	493.10 ± 54.42 ^d^	408.38 ± 12.62 ^c^	208.03 ± 38.15 ^a^	1.45
39	2-Pentylfuran	Fruity, green, earthy, beany, vegetable, metallic	3777-69-3	C_9_H_14_O	138.2	1236.8	505.929	1.2563	2352.86 ± 278.76 ^b^	1509.79 ± 29.49 ^a^	7449.78 ± 728.27 ^d^	3912.17 ± 3.20 ^c^	8375.78 ± 31.18 ^e^	8946.42 ± 232.99 ^f^	1.23
40	α-Pinene	Woody	80-56-8	C_10_H_16_	136.2	1043.9	265.238	1.216	511.38 ± 27.69 ^a^	539.81 ± 3.39 ^a^	880.05 ± 194.93 ^c^	746.14 ± 18.51 ^b^	565.60 ± 51.70 ^a^	1014.47 ± 55.56 ^d^	1.42

MW: molecular weight; RI: retention index; Rt: retention time; Dt: drift time; RIP: reactive ion peak; VIP: variable importance in projection; suffix M represents the monomer of volatile compound and suffix d represents the dimer of volatile compound; a and b, values with different letters in a row indicate significant differences using Duncan’s multiple comparison tests (*p* < 0.05).

**Table 2:** Volatile compounds identified of OAV ≥ 1 via GC-O-MS in yellow horn seed oil.

No.	Compounds	Odor Threshold (mg/kg)	Odor Description	Formula	Content/mg/kg						OAVs						O
					120 °C	130 °C	140 °C	150 °C	160 °C	170 °C	120 °C	130 °C	140 °C	150 °C	160 °C	170 °C	
1	1-Pentanol	0.1502	Sweet, fruity	C_5_H_12_O	0.0256 ± 0.00 ^a^	0.0166 ± 0.00 ^a^	0.0623 ± 0.01 ^ab^	0.1263 ± 0.03 ^cd^	0.1676 ± 0.00 ^d^	0.1017 ± 0.02 ^bc^	<1	<1	<1	<1	1	<1	
2	2-Furanmethanol	0.001	Bitter, spicy, burnt	C_5_H_6_O_2_	0.0061 ± 0.00 ^a^	0.0119 ± 0.00 ^a^	0.0701 ± 0.03 ^c^	0.0662 ± 0.00 ^bc^	0.1614 ± 0.01 ^d^	0.0151 ± 0.00 ^ab^	6	12	70	66	161	15	
3	1-Hexanol	0.0056	Ethereal, fusel oil, fruity, alcoholic, sweet, green	C_6_H_14_O	0.0219 ± 0.00 ^a^	0.0124 ± 0.00 ^a^	0.0985 ± 0.06 ^a^	0.0345 ± 0.00 ^a^	0.117 ± 0.09 ^a^	0.0288 ± 0.02 ^a^	4	2	18	6	21	5	3
4	1-Nonanol	0.0455	Floral, soapy	C_9_H_20_O	0.0048 ± 0.00 ^ab^	0.0019 ± 0.00 ^a^	0.0113 ± 0.00 ^bc^	0.016 ± 0.00 ^c^	0.0457 ± 0.00 ^d^	0.0164 ± 0.00 ^c^	<1	<1	<1	<1	1	<1	
5	1-Heptanol	0.0054	Waxy, woody	C_7_H_16_O	0.0086 ± 0.00 ^ab^	0.0039 ± 0.00 ^a^	0.0165 ± 0.00 ^bc^	0.0249 ± 0.00 ^c^	0.0691 ± 0.00 ^d^	0.0229 ± 0.00 ^c^	2	<1	3	5	13	4	
6	1-Octen-3-ol	0.0015	Mushroom, earthy	C_8_H_16_O	0.0104 ± 0.00 ^ab^	0.0054 ± 0.00 ^a^	0.027 ± 0.00 ^ab^	0.0453 ± 0.01 ^b^	0.1519 ± 0.00 ^c^	0.0433 ± 0.02 ^b^	7	4	18	30	101	29	5
7	n-Butyl acetate	0.058	Ethereal, solvent, fruity, banana	C_6_H_12_O_2_	0.0176 ± 0.00 ^a^	0.0433 ± 0.01 ^ab^	0.0898 ± 0.01 ^bc^	0.1828 ± 0.03 ^d^	0.2605 ± 0.01 ^e^	0.1193 ± 0.02 ^cd^	<1	<1	2	3	4	2	
8	Ethyl 2-methylbutyrate	0.000 013	Fruity	C_7_H_14_O_2_	0.0083 ± 0.00 ^b^	0.0013 ± 0.00 ^a^	0.0003 ± 0.00 ^a^	0.0013 ± 0.00 ^a^	0.0016 ± 0.00 ^a^	ND	638	100	23	100	123	−	3
9	Isoamyl acetate	0.000 15	Sweet, fruity, banana, solvent	C_7_H_14_O_2_	0.0154 ± 0.00 ^b^	0.0019 ± 0.00 ^a^	0.0023 ± 0.00 ^a^	0.0015 ± 0.00 ^a^	0.0015 ± 0.00 ^a^	0.0015 ± 0.00 ^a^	103	13	15	10	10	10	
10	Styrene	0.065	Sweet, balsam, floral, plastic	C_8_H_8_	0.2365 ± 0.03 ^b^	0.0235 ± 0.01 ^a^	0.0087 ± 0.01 ^a^	0.0209 ± 0.01 ^a^	0.0512 ± 0.00 ^a^	0.0084 ± 0.00 ^a^	4	<1	<1	<1	<1	<1	2
11	p-Cymene	0.00501	Citrus	C_10_H_14_	0.0106 ± 0.00 ^b^	0.0065 ± 0.00 ^ab^	0.01 ± 0.00 ^b^	0.0116 ± 0.00 ^bc^	0.0171 ± 0.00 ^c^	0.0035 ± 0.00 ^a^	2	1	2	2	3	<1	
12	Isobutyraldehyde	0.0015	Fresh, aldehydic, floral, green	C_4_H_8_O	0.8988 ± 0.1 ^ab^	0.6502 ± 0.32 ^ab^	0.2576 ± 0.2 ^ab^	0.5103 ± 0.4 ^ab^	1.1364 ± 0.06 ^b^	0.0072 ± 0.00 ^a^	599	433	172	340	758	5	
13	3-Methylbutanal	0.0011	Ethereal, aldehydic, chocolate, peach, fatty	C_5_H_10_O	0.0029 ± 0.00 ^ab^	0.0034 ± 0.00 ^ab^	0.0039 ± 0.00 ^ab^	0.0061 ± 0.00 ^bc^	0.0084 ± 0.00 ^c^	0.0015 ± 0.00 ^a^	3	3	4	6	8	1	3
14	Pentanal	0.012	Fermented, bready, fruity, nutty, berry	C_5_H_10_O	0.0059 ± 0.00 ^a^	0.0031 ± 0.00 ^a^	0.0139 ± 0.00 ^ab^	0.0294 ± 0.01 ^bc^	0.0449 ± 0.00 ^c^	0.0279 ± 0.00 ^b^	<1	<1	1	2	4	2	2
15	Hexanal	0.005	Fresh, green, fatty, aldehydic, grass, leafy, fruity, sweaty	C_6_H_12_O	0.1919 ± 0.03 ^bc^	0.0435 ± 0.01 ^a^	0.0898 ± 0.01 ^a^	0.1823 ± 0.03 ^bc^	0.2607 ± 0.01 ^c^	0.1193 ± 0.02 ^ab^	38	9	18	36	52	24	5
16	Heptanal	0.0028	Fresh, aldehydic, fatty, green, herbal, wine-lee, ozone	C_7_H_14_O	0.0039 ± 0.00 ^a^	0.0015 ± 0.00 ^a^	0.0121 ± 0.00 ^ab^	0.0229 ± 0.00 ^bc^	0.0275 ± 0.00 ^c^	0.0122 ± 0.00 ^ab^	1	<1	4	8	10	4	
17	Octanal	0.000 587	Aldehydic, waxy, citrus, orange, peel, green, herbal, fresh, fatty	C_8_H_16_O	0.008 ± 0.00 ^ab^	0.004 ± 0.00 ^a^	0.0126 ± 0.00 ^abc^	0.0182 ± 0.00 ^bc^	0.07 ± 0.00 ^d^	0.024 ± 0.00 ^c^	14	7	21	31	119	41	
18	2-Octenal (isomer)	0.003	Fresh, cucumber, fatty, green, herbal, banana, waxy, leaf	C_8_H_14_O	0.0085 ± 0.00 ^a^	0.0015 ± 0.00 ^a^	0.0022 ± 0.00 ^a^	0.0136 ± 0.01 ^a^	0.0101 ± 0.00 ^a^	0.0063 ± 0.00 ^a^	3	<1	<1	5	3	2	5
19	Nonanal	0.0011	Waxy, aldehydic, rose, fresh, orris, orange, peel, fatty, peel	C_9_H_18_O	0.0275 ± 0.00 ^a^	0.0106 ± 0.00 ^a^	0.0383 ± 0.01 ^a^	0.0495 ± 0.01 ^a^	0.2198 ± 0.03 ^b^	0.1854 ± 0.05 ^b^	25	10	35	45	200	169	3
20	(E)-2,4-Decadienal	0.000 3	Fried	C_10_H_16_O	0.0026 ± 0.00 ^ab^	0.0014 ± 0.00 ^a^	0.0025 ± 0.00 ^ab^	0.0019 ± 0.00 ^ab^	0.0055 ± 0.00 ^bc^	0.0066 ± 0.00 ^c^	9	5	8	6	18	22	
21	(E, E)-2,4-Decadienal	0.000 027	Earthy, fried, oily	C_10_H_16_O	0.0008 ± 0.00 ^a^	0.0003 ± 0.00 ^a^	0.0016 ± 0.00 ^a^	0.0023 ± 0.00 ^a^	0.0118 ± 0.00 ^b^	0.0167 ± 0.00 ^b^	30	11	59	85	437	619	2
22	Acetoin	0.014	Sweet, buttery, creamy, dairy, milky, fatty	C_4_H_8_O_2_	0.0026 ± 0.00 ^ab^	0.0017 ± 0.00 ^a^	0.0323 ± 0.00 ^a^	0.0072 ± 0.00 ^a^	0.0801 ± 0.00 ^a^	0.0047 ± 0.00 ^a^	<1	<1	2	<1	6	<1	
23	2-Octanone	0.0502	Herbal	C_8_H_16_O	0.0037 ± 0.00 ^a^	0.0035 ± 0.00 ^a^	0.0194 ± 0.00 ^b^	0.027 ± 0.00 ^c^	0.062 ± 0.00 ^d^	0.0139 ± 0.00 ^b^	<1	<1	<1	<1	1	<1	
24	4-hydroxy-2,5-Dimethyl-3(2H)-furanone	0.0223	Sweet, soap, bread	C_6_H_8_O_3_	0.005 ± 0.00 ^a^	0.0132 ± 0.01 ^a^	0.0305 ± 0.01 ^a^	0.0507 ± 0.00 ^ab^	0.0236 ± 0.01 ^c^	0.0109 ± 0.00 ^b^	<1	<1	1	2	1	<1	2
25	2-Decanone	0.0083	−	C_10_H_20_O	0.0023 ± 0.00 ^a^	0.0013 ± 0.00 ^a^	0.0056 ± 0.00 ^a^	0.0063 ± 0.00 ^a^	0.029 ± 0.00 ^c^	0.0129 ± 0.00 ^b^	<1	<1	<1	<1	3	2	
26	2,5-Dimethylpyrazine	1.75	Roasted, nutty, popcorn	C_6_H_8_N_2_	0.4802 ± 0.05 ^a^	0.3186 ± 0.08 ^a^	0.7016 ± 0.18 ^b^	0.9698 ± 0.03 ^ab^	2.7837 ± 0.1 ^c^	0.7895 ± 0.17 ^ab^	<1	<1	<1	<1	2	<1	5
27	2-Ethyl-6-Methylpyrazine	0.04	Roasted, baked potato	C_7_H_10_N_2_	0.0125 ± 0.00 ^ab^	0.0078 ± 0.00 ^a^	0.0259 ± 0.01 ^ab^	0.0324 ± 0.00 ^b^	0.1012 ± 0.00 ^c^	0.0213 ± 0.00 ^ab^	<1	<1	<1	<1	3	<1	
28	2,3,5-Trimethylpyrazine	0.35012	Roasted, nutty	C_7_H_10_N_2_	0.1904 ± 0.02 ^ab^	0.1191 ± 0.03 ^a^	0.1909 ± 0.05 ^ab^	0.3026 ± 0.01 ^b^	0.7995 ± 0.03 ^c^	0.2317 ± 0.05 ^ab^	<1	<1	<1	<1	2	<1	4
29	3-Ethyl-2,5-Dimethylpyrazine	0.0086	roasted, nutty	C_8_H_12_N_2_	0.0615 ± 0.01 ^ab^	0.0326 ± 0.01 ^a^	0.0547 ± 0.02 ^ab^	0.1023 ± 0.00 ^b^	0.2721 ± 0.01 ^c^	0.0859 ± 0.02 ^ab^	7	4	6	12	32	10	4
30	2-Ethyl-3,5-Dimethylpyrazine	0.000 04	Nutty, roasted, sweet	C_8_H_12_N_2_	0.0062 ± 0.00 ^ab^	0.0038 ± 0.00 ^a^	0.006 ± 0.00 ^ab^	0.011 ± 0.00 ^b^	0.0348 ± 0.00 ^c^	0.0104 ± 0.00 ^ab^	155	95	150	275	870	260	
31	2,6-Diethylpyrazine	0.006	Roasted, nutty, sweet	C_8_H_12_N_2_	0.0076 ± 0.00 ^a^	0.0043 ± 0.00 ^a^	0.0047 ± 0.00 ^ab^	0.0109 ± 0.00 ^a^	0.0324 ± 0.00 ^b^	0.0094 ± 0.00 ^a^	1	<1	<1	2	5	2	
32	2,3-Diethyl-5-Methylpyrazine	0.000 031	Raw peanut, potato	C_9_H_14_N_2_	0.0009 ± 0.00 ^ab^	0.0008 ± 0.00 ^a^	0.0013 ± 0.00 ^ab^	0.0027 ± 0.00 ^b^	0.0068 ± 0.00 ^c^	0.0021 ± 0.00 ^ab^	29	26	42	87	219	68	
33	2-Pentylfuran	0.0058	Fruity, green, earthy, beany, vegetable, metallic	C_9_H_14_O	0.0079 ± 0.00 ^a^	0.0029 ± 0.00 ^a^	0.0214 ± 0.01 ^a^	0.0259 ± 0.00 ^a^	0.1252 ± 0.00 ^b^	0.0349 ± 0.01 ^a^	1	<1	4	4	22	6	2
34	1-Methylpyrrolidine	0.0167	−	C_5_H_11_N	0.0002 ± 0.00 ^a^	0.0031 ± 0.00 ^a^	0.0072 ± 0.00 ^b^	0.0039 ± 0.00 ^b^	0.0245 ± 0.01 ^c^	0.0058 ± 0.00 ^a^	<1	<1	<1	<1	1	<1	1
35	2-Pentylpyridine	0.000 6	−	C_10_H_15_N	0.0038 ± 0.00 ^a^	0.0011 ± 0.00 ^ab^	0.006 ± 0.00 ^c^	0.0064 ± 0.00 ^bc^	0.0554 ± 0.01 ^d^	0.0232 ± 0.01 ^abc^	6	2	10	11	92	39	3

ND: not detected; OAVs: ratio of compound concentration to corresponding odor threshold; O: aroma level; a and b: values with different letters in a row indicated significant differences using Duncan’s multiple comparison tests (*p* < 0.05).

## Data Availability

The data used to support the findings of this study can be made available by the corresponding author upon request.

## Supplementary Materials

The following supporting information can be downloaded at: https://www.mdpi.com/article/10.3390/foods12173174/s1, Figure S1: Rose and pie chart of VOCs detected by GC-IMS (A), (B) and GC-O-MS(C), (D) in yellow horn seed oil; Figure S2: Pie chart(A), heat map (B), and VIP map (C) of 24 VOCs detected jointly by GC-MS and GC-IMS; Table S1: The VOCs identified by GC-IMS in yellow horn seed oil; Table S2: The VOCs identified by GC-O-MS in yellow horn seed oil; Table S3: The VOCs identified by the combination of olfactory time and GC-MS retention time.
