# Supplementary material for: Comparative Analysis of Key Odorants and Aroma Characteristics in Hot-Pressed Yellow Horn (Xanthoceras sorbifolia bunge) Seed Oil Via Gas Chromatography–Ion Mobility Spectrometry and Gas Chromatography–Olfactory-Mass Spectrometry
Source: Foods. 2023 Aug 23;12(17):3174. doi: 10.3390/foods12173174 (PMC10487206; doi:10.3390/foods12173174)
Supplement: Supplementary file 1 [file foods-12-03174-s001.zip › foods-2515557-supplementary/foods-2515557-supplementary.pdf]

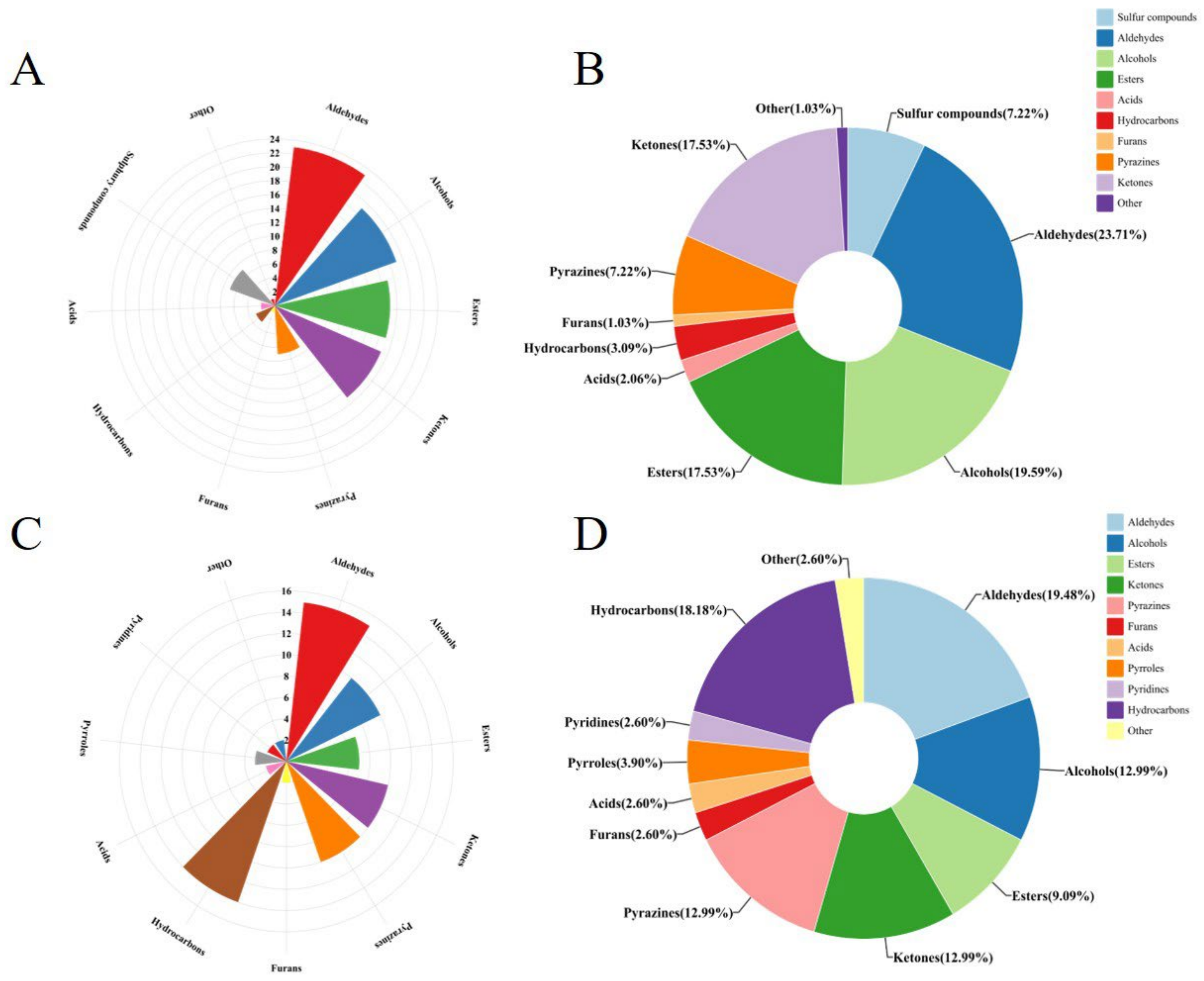

Figure S1. Rose and pie chart of VOCs detected by GC-IMS (A), (B) and GC-O-MS(C), (D) in yellow horn seed oil.

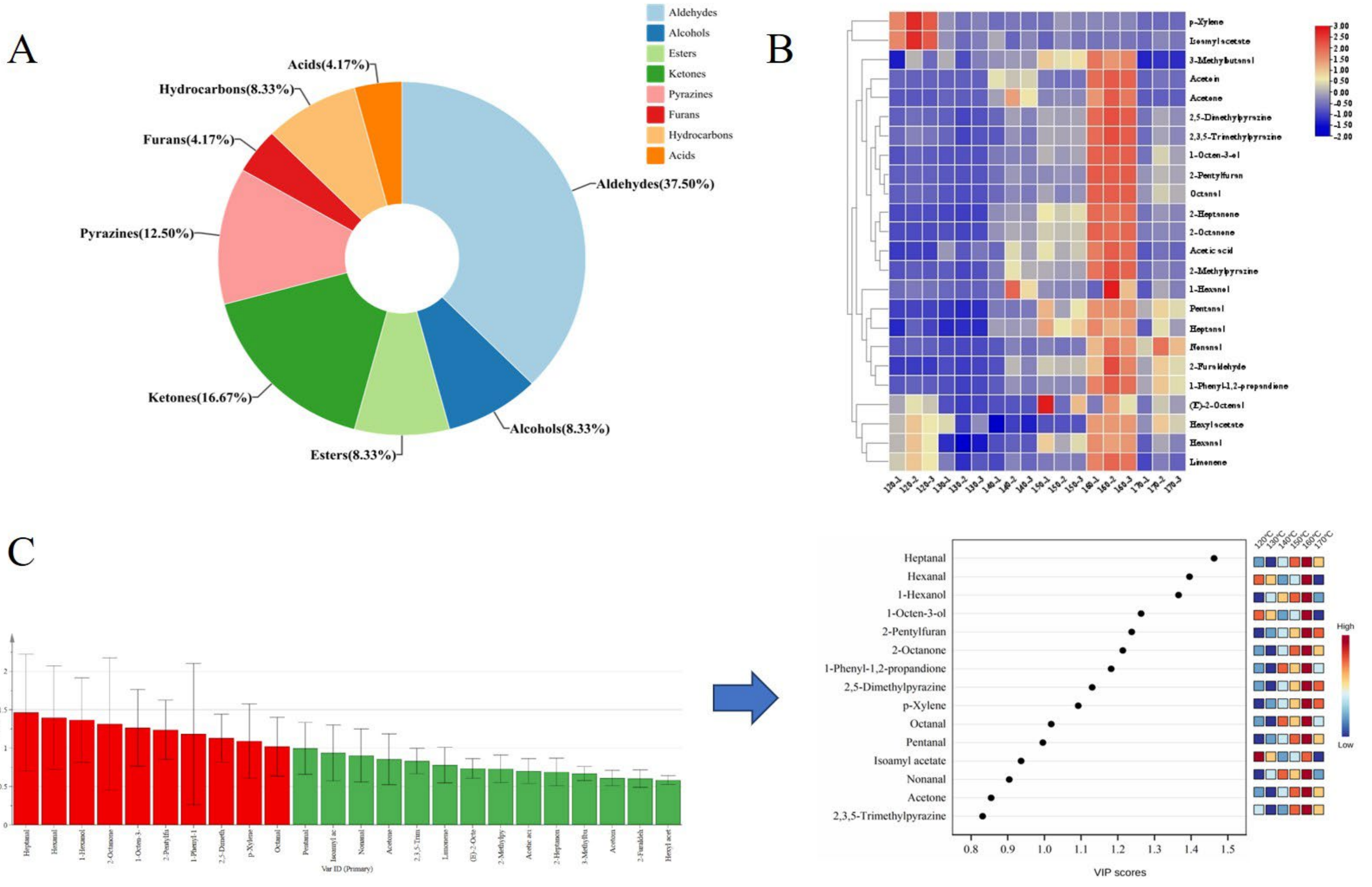

Figure S2. Pie chart(A), heat map (B), and VIP map (C) of 24 VOCs detected jointly by GC-MS and GC-IMS.

Table S1. The VOCs identified by GC-IMS in yellow horn seed oil.

NO	Compounds	Odor description	CAS	Formula	MW	RI	Rt[sec]	Dt[RIP rel]	Peak intensities						VIP
									120℃	130℃	140℃	150℃	160℃	170℃	
1	Allyl methyl sulfide	Sulfurous	10152-76-8	C_4H_8S	88.2	956.4	212.524	1.0444	8057.49±57.43^{b}	9167.78±246.58^{c}	8105.53±580.62^{b}	6730.20±90.04^{a}	5993.88±137.32^{a}	6654.80±140.59^{a}	1.19
2	Dimethyl disulfide	Moldy, onion-like, putrid, unpleasant, cabbage-like	624-92-0	$C_2H_6S_2$	94.2	1095.1	308.773	1.1483	52366.17±3599.12^{b}	23361.85±1372.65^{a}	47382.14±5800.59^{b}	46471.18±543.85^{b}	71141.45±3969.37^{c}	39825.86±3902.28^{b}	1.22
3	Allyl sulfide	Garlic-horseradish like, pungent, sweet	592-88-1	$C_6H_{10}S$	114.2	1117.7	331.892	1.1238	335.37±42.21^{a}	522.73±8.70^{b}	473.84±54.80^{b}	523.42±6.21^{b}	549.46±23.99^{b}	746.14±22.86^{c}	0.87
4	Dimethyl trisulfide	Sulphury, cabbage	3658-80-8	$C_2H_6S_3$	126.3	1412.7	961.969	1.2937	540.84±31.16^{a}	497.46±22.96^{a}	760.40±50.73^{c}	618.64±14.33^{b}	638.54±43.64^{b}	882.27±19.98^{d}	1.21
5	Diethyl disulfide	Onion, moldy, sulfur	110-81-6	$C_4H_{10}S_2$	122.2	1186.5	421.014	1.1476	10791.32±952.54^{cd}	4143.10±316.85^{a}	9578.81±909.06^{bc}	8562.63±402.53^{bc}	12593.85±454.33^{d}	7971.58±694.98^{b}	1.15
6	2-Furfurylthiol	Coffee-like	98-02-2	C_5H_6OS	114.2	1399.6	917.063	1.1071	5801.82±65.94^{a}	6862.40±134.06^{b}	14499±1525.19^{f}	9227.04±77.74^{d}	10126.39±174.88^{e}	8020.17±153.08^{c}	1.58
7	Dipropyl disulfide	Burnt green onion	629-19-6	$C_6H_{14}S_2$	150.3	1357.4	786.141	1.2629	548.27±17.94^{b}	473.63±16.81^{a}	575.04±80.25^{b}	878.77±11.82^{c}	1080.74±23.25^{d}	1955.57±43.09^{e}	0.80
8	2-Furaldehyde	Sweet, woody, almond, fragrant, baked, bread	98-01-1	$C_5H_4O_2$	96.1	1469.7	1184.742	1.0923	5533.32±216.26^{a}	6832.09±533.28^{b}	11350.43±1214.86^{c}	12646.97±276.81^{d}	15050.90±415.74^{e}	18173.29±358.30^{f}	0.60
9	Propanal-M	Green, malt	123-38-6	C_3H_6O	58.1	776.8	155.876	1.1551	31954.12±925.33^{a}	35180.27±62.91^{b}	38930.74±74.09^{c}	38453.06±60.23^{c}	39841.06±72.93^{d}	40526.91±106.96^{e}	0.72
10	Propanal-D	Green, malt	123-38-6	C_3H_6O	58.1	821.4	165.573	1.0351	3911.49±49.11^{e}	3012.49±88.86^{d}	2597.74±210.33^{c}	2577.03±70.91^{c}	2398.20±41.09^{b}	1953.95±59.44^{a}	0.82
11	Butanal-M	Pungent, cocoa, musty, green, malty, bready	123-72-8	C_4H_8O	72.1	920.5	196.738	1.29	8501.31±178.27^{b}	8043.40±76.51^{a}	6619.18±70.29^{cd}	7157.29±56.58^{bc}	6768.29±153.57^{e}	7544.83±145.73^{de}	1.00
12	Butanal-D	Pungent, cocoa, musty, green, malty, bready	123-72-8	C_4H_8O	72.1	835.1	169.008	1.1041	6507.65±93.70^{e}	5284.81±32.66^{d}	7014.88±635.33^{a}	6792.97±71.16^{b}	7635.27±318.18^{a}	7298.71±109.82^{c}	0.95
13	3-Methylbutanal	Ethereal, aldehydic, chocolate, peach, fatty	590-86-3	$C_5H_{10}O$	86.1	905.8	191.09	1.4114	12217.41±318.87^{e}	11507.65±143.28^{d}	7543.68±227.72^{b}	8623.09±62.95^{c}	7333.47±187.31^{b}	6469.99±35.74^{a}	0.72
14	Hexanal-M	Fresh, green, fatty, aldehydic, grass,	66-25-1	$C_6H_{12}O$	100.2	1096	309.662	1.5677	7780.20±1173.47^{a}	9160.00±228.77^{b}	10374.69±2078.82^{b}	10037.92±274.70^{ab}	8993.22±430.72^{ab}	13609.09±407.11^{ab}	1.04

		leafy, fruity, sweaty												
15	Hexanal-D	Fresh, green, fatty, aldehydic, grass, leafy, fruity, sweaty	66-25-1	$C_6H_{12}O$	100.2	1094.6	308.338	1.2575	11718.59±1742.32^{a}	13205.83±223.23^{ab}	13222.23±788.05^{b}	12805.39±134.00^{b}	12532.19±152.99^{ab}	12756.45±136.71^{c}	1.32
16	(E)-2-Pentenal	Pungent, green, fruity, apple, orange, tomato	1576-87-0	C_5H_8O	84.1	1143.8	362.171	1.1088	6509.70±1184.66^{a}	10950.67±883.95^{c}	6262.04±311.96^{a}	6272.97±491.38^{a}	6794.17±455.91^{a}	8605.50±479.22^{b}	1.41
17	Heptanal-M	Fresh, aldehydic, fatty, green, herbal, wine-lee, ozone	111-71-7	$C_7H_{14}O$	114.2	1195.1	434.412	1.3307	4318.85±670.22^{a}	7012.96±1232.49^{a}	4257.53±460.87^{a}	4610.66±255.91^{b}	4856.44±198.56^{c}	6485.02±371.22^{d}	1.25
18	Heptanal-D	Fresh, aldehydic, fatty, green, herbal, wine-lee, ozone	111-71-7	$C_7H_{14}O$	114.2	1195.1	434.332	1.6976	187.54±36.10^{a}	157.56±17.03^{b}	171.14±5.43^{a}	242.62±33.33^{a}	303±24.52^{a}	615.48±30.67^{b}	0.84
19	Pentanal-M	Fermented, bready, fruity, nutty, berry	110-62-3	$C_5H_{10}O$	86.1	986.9	228.322	1.4232	17994.72±780.36^{a}	14804.77±219.54^{a}	24647.98±835.88^{d}	26989.3±65.46^{c}	26647.45±43.91^{b}	32551.66±84.12	0.83
20	Pentanal-D	Fermented, bready, fruity, nutty, berry	110-62-3	$C_5H_{10}O$	86.1	997.2	234.237	1.183	1109.94±143.23^{b}	1186.72±32.15^{a}	1778.92±40.76^{c}	1750.27±45.62^{d}	1633.28±18.72^{d}	1358.12±9.39^{e}	0.98
21	(E)-2-Octenal	Fresh, cucumber, fatty, green, herbal, banana, waxy, leaf	2548-87-0	$C_8H_{14}O$	126.2	1390.5	887.05	1.3391	650.49±53.84^{a}	1384.16±58.72^{b}	5060.08±119.39^{c}	5942.22±41.35^{d}	6175.79±111.47^{e}	8800.56±119.31^{f}	0.64
22	Nonanal	Waxy, aldehydic, rose, fresh, orris, orange, peel, fatty, peely	124-19-6	$C_9H_{18}O$	142.2	1350.6	766.75	1.4718	1835.33±37.75^{b}	1272.63±72.01^{a}	1310.59±437.87^{a}	2112.54±79.44^{c}	3326.76±53.35^{d}	5646.14±37.25^{e}	0.86
23	(E)-2-Heptenal-M	Pungent, green, vegetable, fresh, fatty	18829-55-5	$C_7H_{12}O$	112.2	1314.7	672.372	1.2551	4828.94±171.63^{a}	6254.27±159.55^{c}	5622.69±107.43^{b}	6434.55±143.6^{c}	7338.08±158^{d}	10029.46±90.77^{e}	0.84
24	(E)-2-Heptenal-D	Pungent, green, vegetable, fresh, fatty	18829-55-5	$C_7H_{12}O$	112.2	1313.3	668.925	1.6717	438.10±68.18^{a}	705.06±58.64^{b}	1029.41±32.25^{c}	1138.21±28.81^{d}	1825.60±36.33^{e}	4004.58±64.02^{f}	0.80
25	(E)-2-Hexenal-M	Green, banana, aldehydic, fatty, cheesy	6728-26-3	$C_6H_{10}O$	98.1	1227	488.02	1.1831	2930.33±291.68^{a}	5069.94±94.47^{a}	6226.68±284.79^{b}	7518.53±219.30^{c}	7692.83±19.99^{d}	11115.32±252.78^{e}	0.69
26	(E)-2-Hexenal-D	Green, banana, aldehydic, fatty, cheesy	6728-26-3	$C_6H_{10}O$	98.1	1226.8	487.721	1.5221	157.99±24.78^{a}	173.96±19.33^{b}	351.38±1.63^{c}	435.11±23.87^{d}	711.89±16.51^{d}	2279.07±64.66^{e}	0.86

27	3-(Methylthio)propanal	Boiled potato	3268-49-3	C_4H_8OS	104.2	1452.5	1112.561	1.0945	2890.02±119.99^{a}	3623.10±19.08^{b}	3983.14±286.23^{c}	4233.28±70.10^{d}	4569.41±113.19^{e}	5973.56±184.63^{f}	0.73
28	Benzaldehyde	Strong, sharp, sweet, bitter, almond, cherry	100-52-7	C_7H_6O	106.1	1469.1	1182.187	1.4699	1458.55±416.72^{bcd}	988.42±194.81^{a}	1227.33±92.57^{abc}	1059.22±99.00^{ab}	1519.44±95.97^{cd}	1792.55±209.82^{d}	1.12
29	(E,E)-2,4-Heptadienal	Fatty, green, oily, aldehydic, vegetable, cake, cinnamon	4313-03-5	$C_7H_{10}O$	110.2	1452.4	1112.091	1.2001	3859.48±455.45^{c}	1762.31±443.30^{ab}	1484.30±338.59^{ab}	1720.04±30.69^{ab}	2233.30±26.19^{b}	3426.50±157.52^{c}	1.21
30	Octanal	Aldehydic, waxy, citrus, orange, peel, green, herbal, fresh, fatty	124-13-0	$C_8H_{16}O$	128.2	1313.9	670.399	1.407	572.35±60.76^{b}	495.32±58.00^{a}	1166.22±24.64^{e}	816.25±37.78^{c}	1045.3±6.54^{d}	1165.11±24.56^{e}	1.13
31	1-Octen-3-ol	Mushroom, earthy	3391-86-4	$C_8H_{16}O$	128.2	1467.6	1175.894	1.1584	40475.67±3853.92^{c}	19298.01±3456.23^{b}	20171.27±4552.03^{b}	13541.02±852.27^{a}	14032.07±158.17^{a}	11865.48±350.39^{a}	1.09
32	Linalool oxide	Musty, camphor, fenchyl, alcohol	60047-17-8	$C_{10}H_{18}O_2$	172.3	1441.1	1069.247	3.2598	9670.70±3053.56^{b}	3190.54±1158.91^{a}	4354.89±332.61^{a}	3202.59±56.06^{a}	3747.35±95.80^{a}	1785.63±101.36^{a}	1.00
33	1-Propanol-M	Fermented	71-23-8	C_3H_8O	60.1	1046.3	267.072	1.1112	4436.79±1190.25^{a}	6565.21±172.18^{cd}	4900.08±461.96^{ab}	5768.09±73.02^{bc}	4305.70±216.30^{a}	6820.04±221.51^{d}	1.21
34	1-Propanol-D	Fermented	71-23-8	C_3H_8O	60.1	1004	238.277	1.245	1268.19±211.08^{a}	1919.45±35.26^{c}	1240.14±112.86^{a}	1705.44±46.12^{b}	1650.61±56.70^{b}	2761.58±52.64^{d}	0.99
35	1-Butanol-M	Fusel oil, sweet, balsam, whiskey	71-36-3	$C_4H_{10}O$	74.1	1154	375.02	1.185	3744.79±598.76^{a}	4548.49±70.81^{b}	4686.03±40.04^{bc}	5376.44±93.79^{d}	4730.05±141.61^{bc}	5125.45±171.36^{cd}	1.25
36	1-Butanol-D	Fusel oil, sweet, balsam, whiskey	71-36-3	$C_4H_{10}O$	74.1	1155.3	376.74	1.377	526.58±109.59^{a}	783.80±5.40^{bc}	705.70±152.36^{b}	902.93±21.41^{c}	881.07±61.10^{c}	1359.14±59.78^{d}	0.83
37	2-Propanol-M	Alcohol, musty, woody	67-63-0	C_3H_8O	60.1	923.6	197.998	1.2259	568.59±43.62^{d}	627.18±6.66^{c}	695.20±107.43^{a}	645.45±25.72^{b}	675.77±30.70^{b}	901.23±30.31^{cd}	0.98
38	2-Propanol-D	Alcohol, musty, woody	67-63-0	C_3H_8O	60.1	951.8	210.309	1.0803	3130.59±296.88^{a}	2748.43±184.86^{ab}	1158.02±332.61^{b}	1562.82±68.13^{ab}	1561.88±73.44^{b}	2819.65±104.31^{c}	1.01
39	2-Methyl-1-Propanol-M	Cortex	78-83-1	$C_4H_{10}O$	74.1	1104.2	317.826	1.1724	1732.68±65.95^{d}	1659.07±82.18^{d}	591.18±140.40^{b}	464.49±32.80^{ab}	1022.75±133.15^{c}	383.79±30.18^{a}	1.22
40	2-Methyl-1-Propanol-D	Cortex	78-83-1	$C_4H_{10}O$	74.1	1099.5	313.154	1.3587	4540.63±217.19^{b}	3929.00±68.96^{a}	5332.68±71.20^{c}	5254.58±52.46^{c}	6482.37±69.89^{e}	6003.19±192.82^{d}	0.90
41	3-Methyl-2-Butanol	Fruity	598-75-4	$C_5H_{12}O$	88.1	1117.7	331.949	1.2297	1242.74±51.75^{b}	1040.34±77.97^{a}	1261.27±165.58^{b}	1619.70±40.5^{c}	2594.03±50.09^{d}	2692.83±123.78^{d}	1.03
42	1-Pentanol-M	Sweet, fruity	71-41-0	$C_5H_{12}O$	88.1	1258.2	546.981	1.2531	20055.85±1366.93^{a}	21098.84±340.74^{a}	20146.42±569.03^{a}	19634.23±37.66^{b}	18467.41±55.47^{b}	16879.40±46.59^{c}	0.86

43	1-Pentanol-D	Sweet, fruity	71-41-0	$C_5H_{12}O$	88.1	1258	546.611	1.5096	6814.24±565.97^{cd}	7150.80±147.04^{d}	6900.36±179.89^{cd}	8311.13±18.77^{c}	8208.22±106.53^{b}	9652.34±183.83^{a}	1.07
44	(Z)-3-Hexen-1-ol	Cucumber-like, fatty	928-96-1	$C_6H_{12}O$	100.2	1390.9	888.328	1.2305	4552.76±217.95^{a}	5723.17±214.36^{b}	8585.01±247.47^{d}	8495.76±98.40^{d}	8432.57±54.06^{cd}	8187.55±43.35^{c}	0.73
45	3-Methyl-1-Pentanol	Wine, cocoa	589-35-5	$C_6H_{14}O$	102.2	1311.8	665.308	1.6007	1403.89±109.44^{a}	1510.56±65.66^{a}	2285.94±8.88^{b}	2901.72±59.17^{c}	3944.03±89.18^{d}	5722.82±106.09^{e}	0.70
46	2-Methyl-1-Butanol-M	Alcohol, cocoa	137-32-6	$C_5H_{12}O$	88.1	1215.2	467.535	1.2362	6259.65±476.01^{c}	6748.22±192.83^{d}	5965.62±121.38^{c}	5435.88±117.62^{b}	4631.75±44.33^{a}	4646.61±173.22^{a}	0.87
47	2-Methyl-1-Butanol-D	Alcohol, cocoa	137-32-6	$C_5H_{12}O$	88.1	1195	434.286	1.4677	3365.44±465.36^{cd}	3718.91±159.65^{d}	3108.47±262.32^{abc}	3256.73±82.89^{bc}	2698.04±27.94^{a}	2879.26±99.53^{ab}	1.00
48	1-Hexanol-M	Ethereal, fusel oil, fruity, alcoholic, sweet, green	111-27-3	$C_6H_{14}O$	102.2	1333.4	719.934	1.3277	3652.99±251.84^{e}	2928.45±150.88^{d}	2126.46±137.32^{ab}	2337.14±24.83^{bc}	1968.21±27.93^{a}	2486.17±58.16^{c}	1.03
49	1-Hexanol-D	Ethereal, fusel oil, fruity, alcoholic, sweet, green	111-27-3	$C_6H_{14}O$	102.2	1333.4	719.934	1.6422	3862.64±426.21^{a}	9007.31±1099.47^{b}	8640.73±633.52^{b}	11297.05±243.94^{c}	8925.33±179.25^{b}	3793.13±137.59^{a}	1.41
50	2,3,5-Trimethylpyrazine	Roasted, nutty	14667-55-1	$C_7H_{10}N_2$	122.2	1442.8	1073.771	1.1723	42411.95±3341.16^{d}	28524.97±7840.17^{c}	20722.74±7519.26^{b}	11039.49±1201.64^{a}	8587.48±523.89^{a}	6611.58±535.28^{a}	0.81
51	2-Methylpyrazine-M	Nutty, meaty, roasted	109-08-0	$C_5H_6N_2$	94.1	1274.1	579.823	1.0845	14297.24±545.55^{b}	14058.29±409.24^{a}	22035.38±702.36^{d}	20916.77±225.14^{c}	21037.09±177.11^{e}	19327.05±121.29^{e}	0.81
52	2-Methylpyrazine-D	Nutty, meaty, roasted	109-08-0	$C_5H_6N_2$	94.1	1270.6	572.449	1.3911	3757.51±118.78^{a}	2968.08±193.29^{a}	8312.41±256.52^{d}	7601.63±44.58^{c}	9425.09±142.69^{c}	9303.82±144.09^{b}	0.67
53	2,5-Dimethylpyrazine-M	Roasted, nutty, popcorn	123-32-0	$C_6H_8N_2$	108.1	1332.1	716.66	1.1083	9907.68±109.10^{b}	8125.21±368.56^{a}	9863.44±169.20^{c}	11573.41±210.26^{d}	12995.57±143.80^{e}	13545.55±136.91^{d}	0.99
54	2,5-Dimethylpyrazine-D	Roasted, nutty, popcorn	123-32-0	$C_6H_8N_2$	108.1	1309.4	659.557	1.4993	758.27±31.44^{b}	605.49±17.87^{a}	966.26±22.28^{b}	1200.64±25.78^{c}	1293.21±47.29^{d}	1177.07±14.31^{e}	1.03
55	Ethylpyrazine	Roasted, peanut butter	13925-00-3	$C_6H_8N_2$	108.1	1369.1	820.493	1.1247	1503.81±133.88^{b}	1300.64±73.53^{a}	1955.32±260.15^{c}	2339.11±20.55^{d}	2558.07±30.88^{e}	2822.64±88.98^{f}	0.78
56	2,6-Dimethylpyrazine	Ethereal, cocoa, nutty, roasted, roasted, meaty, beefy, brown, coffee, buttermilk	108-50-9	$C_6H_8N_2$	108.1	1333.1	719.115	1.5327	397.11±10.40^{b}	204.19±14.61^{a}	160.77±34.96^{a}	187.28±8.70^{a}	179.17±37.07^{a}	187.71±26.50^{a}	1.16
57	Triethylenediamine	-	280-57-9	$C_6H_{12}N_2$	112.2	1501.8	1332.119	1.1697	6224.55±1178.89^{c}	2280.61±535.37^{ab}	2968.33±1143.49^{b}	1486.64±121.80^{a}	1365.63±17.25^{a}	1285.78±107.58^{a}	1.09

58	Isopropyl acetate	-	108-21-4	$C_5H_{10}O_2$	102.1	853.5	174.02	1.1686	562.96±140.87^{a}	626.84±38.31^{a}	1008.02±82.79^{b}	1205.85±4.73^{c}	1151.11±24.41^{c}	1017.20±8.84^{b}	0.99
59	Ethyl acetate	Ethereal, fruity, sweet, weedy, green	141-78-6	$C_4H_8O_2$	88.1	902.4	189.853	1.3308	16501.59±119.01^{c}	15731.71±375.26^{b}	15527.64±623.01^{b}	15039.45±117.88^{a}	14789.14±43.39^{a}	14684.96±62.13^{a}	0.85
60	Ethyl isobutyrate	-	97-62-1	$C_6H_{12}O_2$	116.2	975.9	222.306	1.2027	546.82±74.510^{b}	699.68±52.59^{c}	331.52±22.10^{a}	349.2±13.10^{a}	295.74±19.37^{a}	291.39±12.54^{a}	0.79
61	Ethyl butyrate	Fruity	105-54-4	$C_6H_{12}O_2$	116.2	1058.1	276.257	1.2052	2012.62±106.02^{a}	1829.35±26.33^{a}	2960.39±376.09^{c}	3076.36±58.21^{c}	2673.53±166.98^{b}	2848.26±97.28^{bc}	1.18
62	Ethyl propanoate	Banana	105-37-3	$C_5H_{10}O_2$	102.1	931.5	201.258	1.1462	16657.36±1056.61^{e}	15370.46±185.67^{d}	8631.25±20.83^{b}	8107.45±240.52^{b}	7109.04±146.14^{a}	9994.11±333.51^{c}	0.77
63	Ethyl cinnamate	Sweet	103-36-6	$C_{11}H_{12}O_2$	176.2	985.4	227.445	1.3935	5366.02±192.89^{a}	5055.51±113.17^{a}	8593.08±810.33^{c}	8114.62±44.54^{b}	9015.93±70.15^{c}	9658.40±28.12^{d}	0.65
64	Ethyl 2-Methylbutanoate	Berry, sweet	7452-79-1	$C_7H_{14}O_2$	130.2	1020.3	248.674	1.2338	401.81±41.51^{a}	469.19±35.58^{b}	914.51±31.70^{d}	834.70±13.33^{c}	1036.59±25.17^{e}	879.19±7.06^{cd}	0.79
65	Methyl isovalerate	-	556-24-1	$C_6H_{12}O_2$	116.2	1019	247.769	1.2025	990.04±30.02^{a}	1192.95±30.08^{b}	1958.26±89.67^{d}	1858.56±21.67^{c}	1974.11±43.58^{d}	2790.19±53.33^{e}	0.71
66	Isoamyl acetate	Sweet, fruity, banana, solvent	123-92-2	$C_7H_{14}O_2$	130.2	1103.5	317.1	1.2968	396.85±38.83^{a}	359.45±25.95^{a}	597.33±57.43^{b}	665.35±29.26^{c}	1064.00±20.07^{e}	838.2±31.48^{d}	1.12
67	Ethyl valerate	Yeast	539-82-2	$C_7H_{14}O_2$	130.2	1141.7	359.588	1.2642	1375.71±105.83^{b}	941.62±98.52^{a}	2059.72±114.67^{d}	1771.71±57.89^{c}	2266.43±49.31^{e}	2021.59±44.61^{d}	0.86
68	Isoamyl isovalerate	-	659-70-1	$C_{10}H_{20}O_2$	172.3	1313.1	668.589	1.4472	2555.08±259.37^{b}	2242.60±120.54^{a}	3705.29±77.72^{c}	3775.88±54.37^{c}	4660.88±57.13^{e}	4219.70±106.15^{d}	0.75
69	Hexyl acetate	Fruity, green, apple, banana, sweet	142-92-7	$C_8H_{16}O_2$	144.2	1225.6	485.53	1.4146	292.58±33.74^{a}	407.87±37.66^{b}	1546.30±38.95^{d}	1349.66±13.10^{c}	1677.34±74.20^{e}	1960.61±78.79^{f}	0.64
70	(Z)-3-Hexen-1-ol acetate	fresh, fruity	3681-71-8	$C_8H_{14}O_2$	142.2	1300	637.406	1.2983	2279.16±78.54^{b}	1889.73±68.43^{a}	3053.78±267.57^{d}	2778.15±84.63^{c}	2826.48±58.66^{c}	2143.88±59.70^{b}	1.07
71	Ethyl hexanoate	Sweet, fruity, pineapple, waxy, green, banana	123-66-0	$C_8H_{16}O_2$	144.2	1226.9	487.988	1.3283	321.10±31.65^{b}	257.82±27.36^{a}	384.94±19.02^{c}	461.76±34.07^{d}	690.12±37.19^{e}	1178.78±55.16^{f}	0.78
72	Butyl propionate	-	590-01-2	$C_7H_{14}O_2$	130.2	1145	363.651	1.2895	283.61±29.02^{a}	301.97±37.90^{a}	575.43±43.12^{b}	583.37±5.29^{b}	718.9±24.37^{c}	868.43±56.46^{d}	0.61
73	Ethyl lactate	Whey, creamy	97-64-3	$C_5H_{10}O_3$	118.1	1313.4	669.166	1.5483	562.53±102.08^{a}	554.50±22.12^{a}	1687.33±11.23^{e}	1144.36±43.15^{c}	1277.58±64.71^{d}	783.55±58.83^{b}	1.20
74	Ethyl acrylate	-	140-88-5	$C_5H_8O_2$	100.1	1003.1	237.762	1.1292	522.31±15.90^{d}	665.01±46.32^{e}	431.57±27.36^{ab}	419.4±12.80^{a}	469.79±3.59^{bc}	487.12±12.09^{cd}	1.40

75	2-Butanone	Fragrant, fruit, pleasant	78-93-3	C_4H_8O	72.1	890.3	185.553	1.2477	10363.63±299.61^{a}	11488.69±59.49^{b}	28958.41±279.89^{d}	26497.31±44.93^{c}	29790.68±133.63^{e}	29554.98±111.21^{e}	0.65
76	3-Pentanone	Ethereal, acetone	96-22-0	$C_5H_{10}O$	86.1	984.1	226.742	1.3587	1123.35±122.13^{a}	1342.23±96.18^{b}	2868.42±201.45^{c}	2991.73±12.47^{c}	4167.35±73.45^{d}	4248.65±19.04^{d}	0.71
77	2-Pentanone-M	Sweet, fruity, ethereal, wine, banana, woody	107-87-9	$C_5H_{10}O$	86.1	1020.1	248.502	1.116	506.17±59.93^{a}	884.49±53.43^{b}	995.21±181.70^{b}	1009.09±25.34^{b}	1212.94±100.61^{c}	1359.65±53.32^{d}	0.89
78	2-Pentanone-D	Sweet, fruity, ethereal, wine, banana, woody	107-87-9	$C_5H_{10}O$	86.1	1029.4	254.809	1.3681	408.64±87.77^{bc}	346.98±27.84^{ab}	369.95±13.41^{bc}	441.01±57.32^{c}	275.33±16.95^{a}	551.34±22.48^{d}	1.81
79	4-Methyl-2-Pentanone	-	108-10-1	$C_6H_{12}O$	100.2	1024.6	251.512	1.1807	2700.01±329.88^{a}	3277.40±69.74^{bc}	3529.54±108.52^{c}	3502.25±31.06^{c}	3046.05±74.26^{b}	3889.29±72.68^{d}	1.17
80	2,3-Pentanedione	Caramel, buttery, sweet	600-14-6	$C_5H_8O_2$	100.1	1074.4	289.875	1.2225	4558.31±2449.92^{a}	13092.33±308.38^{d}	10324.22±1396.57^{c}	11519.26±235.03^{cd}	8022.99±433.80^{b}	11561.28±335.72^{cd}	1.20
81	2,3-Butanedione	Buttery	431-03-8	$C_4H_6O_2$	86.1	974.8	221.722	1.1692	1109.94±143.23^{a}	1186.72±32.15^{a}	1778.92±40.76^{d}	1750.27±45.62^{d}	1633.28±18.72^{c}	1358.12±9.39^{b}	0.98
82	2-Hexanone	Soapy, banana	591-78-6	$C_6H_{12}O$	100.2	1071.2	287.036	1.1908	656.73±128.08^{a}	647.08±76.99^{a}	1114.34±277.36^{b}	1527.29±38.34^{c}	1425.84±144.80^{c}	1041.54±26.66^{b}	1.30
83	1-Hydroxypropan-2-one-M	Berry, sweet	116-09-6	$C_3H_6O_2$	74.1	1301.8	641.394	1.0434	13614.72±791.77^{a}	18528.55±693.32^{d}	16564.74±415.40^{b}	17575.49±100.62^{c}	16455.47±62.10^{b}	13047.75±275.54^{a}	1.34
84	1-Hydroxypropan-2-one-D	Berry, sweet	116-09-6	$C_3H_6O_2$	74.1	1287.8	609.588	1.2362	16409.52±1643.48^{b}	16502.61±467.65^{b}	15641.14±1495.48^{b}	18870.41±302.10^{c}	16271.60±197.92^{b}	7496.5±368.51^{a}	1.32
85	3-Hydroxy-2-Butanone-M	Sweet, buttery, creamy, dairy, milky, fatty	513-86-0	$C_4H_8O_2$	88.1	1288.6	611.186	1.0539	17215.02±545.67^{e}	17331.42±477.57^{d}	8956.88±152.36^{c}	9956.62±61.61^{c}	7480.36±128.19^{b}	4506.56±92.27^{a}	0.62
86	3-Hydroxy-2-Butanone-D	Sweet, buttery, creamy, dairy, milky, fatty	513-86-0	$C_4H_8O_2$	88.1	1287.7	609.321	1.336	8160.57±316.97^{e}	6715.34±333.80^{e}	5116.31±45.65^{c}	5032.96±34.75^{d}	4637.48±100.06^{b}	2982.34±45.97^{a}	0.69
87	2-Heptanone-M	Fruity, spicy, sweet, herbal, coconut, woody	110-43-0	$C_7H_{14}O$	114.2	1190.8	427.68	1.2622	2639.97±291.91^{a}	2429.46±108.82^{a}	6642.63±970.84^{c}	6213.28±50.34^{bc}	6661.29±60.33^{c}	5911.05±82.34^{b}	0.73
88	2-Heptanone-D	Fruity, spicy, sweet, herbal, coconut, woody	110-43-0	$C_7H_{14}O$	114.2	1191.4	428.582	1.6324	159.70±22.33^{a}	147.32±18.70^{a}	468.21±61.05^{b}	894.57±34.30^{c}	1025.14±82.80^{d}	1536.77±64.56^{e}	0.79
89	3-Octanone	-	106-68-3	$C_8H_{16}O$	128.2	1278.4	589.018	1.3106	186.09±36.02^{a}	177.38±16.40^{a}	401.59±37.14^{b}	425.55±15.19^{b}	670.99±9.67^{c}	1136.5±37.43^{d}	0.74
90	2-Octanone	Herbal	111-13-7	$C_8H_{16}O$	128.2	1314.2	671.249	1.3332	248.09±23.85^{ab}	297.45±9.64^{b}	822.91±100.36^{e}	493.1±54.42^{d}	408.38±12.62^{c}	208.03±38.15^{a}	1.45

91	Acetone	Solvent, ethereal, apple, pear	67-64-1	C_3H_6O	58.1	813.1	163.59	1.1184	319.82±48.53[a]	666.63±43.02[b]	2702.53±237.86[d]	2523.14±61.02[cd]	2962.87±159.34[e]	2392.91±50.99[c]	0.70
92	2-Pentylfuran	Fruity, green, earthy, beany, vegetable, metallic	3777-69-3	$C_9H_{14}O$	138.2	1236.8	505.929	1.2563	2352.86±278.76[b]	1509.79±29.49[a]	7449.78±728.27[d]	3912.17±3.20[c]	8375.78±31.18[e]	8946.42±232.99[f]	1.23
93	Acetic acid-M	Vinegar	64-19-7	$C_2H_4O_2$	60.1	1405	935.275	1.0508	121433.25±10341.87[d]	111133.75±558.49[d]	103735.89±668.12[bc]	88474.57±1210.85[c]	89535.15±611.36[ab]	69723.29±2418.56[a]	0.78
94	Acetic acid-D	Vinegar	64-19-7	$C_2H_4O_2$	60.1	1405	935.275	1.0508	138019.55±6377.67[d]	136049.20±707.69[c]	123394.25±7296.61[c]	125368.82±1316.19[b]	118859.72±1741.12[b]	113372.94±592.64[a]	0.71
95	α-Pinene	Woody	80-56-8	$C_{10}H_{16}$	136.2	1043.9	265.238	1.216	511.38±27.69[a]	539.81±3.39[a]	880.05±194.93[c]	746.14±18.51[b]	565.60±51.70[a]	1014.47±55.56[d]	1.42
96	p-Xylene	Plastic	106-42-3	C_8H_{10}	106.2	1143.1	361.262	1.0748	773.81±85.55[a]	899.35±53.69[a]	1207.21±199.64[c]	1153.93±54.16[bc]	1055.97±56.75[b]	1108.66±19.22[bc]	0.95
97	Limonene	Lemon, citrus	138-86-3	$C_{10}H_{16}$	136.2	1242.6	516.677	1.2272	208.80±46.72[a]	196.42±12.35[a]	431.18±21.01[b]	778.68±45.24[c]	1101.92±12.27[d]	1763.68±42.23[e]	0.74

MW: Molecular weight; RI: Retention index; Rt: Retention time; Dt: Drift time; RIP: Reactive ion peak; VIP: Variable importance in projection; Suffix M represented the monomer of volatile compound and suffix D represented the dimer of volatile compound; Values with different letters in a row indicated significant differences using Duncan's multiple comparison tests ($p < 0.05$).

Table S2. The VOCs identified by GC-O-MS in yellow horn seed oil.

NO	Compounds	Odor threshold （mg/Kg）	Odor description	CAS	Formula	Content/mg/Kg						OAV					
						120℃	130℃	140℃	150℃	160℃	170℃	120℃	130℃	140℃	150℃	160℃	170℃
	Alcohols																
1	1-Pentanol	0.1502	Sweet, fruity	71-41-0	$C_5H_{12}O$	0.0256±0.00^{a}	0.0166±0.00^{a}	0.0623±0.01^{ab}	0.1263±0.03^{cd}	0.1676±0.00^{d}	0.1017±0.02^{bc}	<1	<1	<1	<1	1	<1
2	2,3-Butanediol	100	Fruity, creamy, buttery	513-85-9	$C_4H_{10}O_2$	0.1648±0.02^{b}	0.0028±0.00^{a}	0.0468±0.03^{a}	0.1943±0.04^{b}	0.1914±0.00^{b}	0.0987±0.02^{ab}	<1	<1	<1	<1	<1	<1
3	2-Furanmethanol	0.001	Bitter, spicy, burnt	98-00-0	$C_5H_6O_2$	0.0061±0.00^{a}	0.0119±0.00^{a}	0.0701±0.03^{c}	0.0662±0.00^{bc}	0.1614±0.01^{d}	0.0151±0.00^{ab}	6	12	70	66	161	15
4	1-Hexanol	0.0056	Ethereal, fusel oil, fruity, alcoholic, sweet, green	111-27-3	$C_6H_{14}O$	0.0219±0.00^{a}	0.0124±0.00^{a}	0.0985±0.06^{a}	0.0345±0.00^{a}	0.117±0.09^{a}	0.0288±0.02^{a}	4	2	18	6	21	5
5	1-Nonanol	0.0455	Floral, soapy	143-08-8	$C_9H_{20}O$	0.0048±0.00^{ab}	0.0019±0.00^{a}	0.0113±0.00^{bc}	0.016±0.00^{c}	0.0457±0.00^{d}	0.0164±0.00^{c}	<1	<1	<1	<1	1	<1
6	1-Heptanol	0.0054	Waxy, woody	111-70-6	$C_7H_{16}O$	0.0086±0.00^{ab}	0.0039±0.00^{a}	0.0165±0.00^{bc}	0.0249±0.00^{c}	0.0691±0.00^{d}	0.0229±0.00^{c}	2	<1	3	5	13	4
7	1-Octen-3-ol	0.0015	Mushroom, earthy	3391-86-4	$C_8H_{16}O$	0.0104±0^{ab}	0.0054±0^{a}	0.027±0^{ab}	0.0453±0.01^{b}	0.1519±0^{c}	0.0433±0.02^{b}	7	4	18	30	101	29
8	2-Ethyl-1-hexanol	25.4822	Citrus, fresh, floral, oily, sweet	104-76-7	$C_8H_{18}O$	0.0013±0^{a}	0.0028±0.00^{a}	0.0052±0.00^{a}	0.0036±0.00^{a}	0.025±0.01^{b}	0.0033±0.00^{a}	<1	<1	<1	<1	<1	<1
9	1-Octanol	0.1258	Soapy, oily	111-87-5	$C_8H_{18}O$	0.0171±0.00^{a}	0.0105±0.01^{a}	0.0102±0.00^{a}	0.0247±0.00^{a}	0.0518±0.01^{b}	0.0317±0.01^{ab}	<1	<1	<1	<1	<1	<1
10	2-Phenylethanol	0.56423	Floral	60-12-8	$C_8H_{10}O$	0.0146±0.00^{a}	0.011±0.00^{a}	0.0132±0.00^{a}	0.0159±0.00^{a}	0.0317±0.00^{b}	0.0077±0.00^{a}	<1	<1	<1	<1	<1	<1
	Esters																
11	Methyl acetate	1.5	Fruity	79-20-9	$C_3H_6O_2$	0.0004±0.00^{a}	0.0019±0.00^{a}	0.006±0.00^{a}	0.0191±0.01^{a}	0.0158±0.00^{a}	0.0109±0.00^{a}	<1	<1	<1	<1	<1	<1
12	n-Butyl acetate	0.058	Ethereal, solvent, fruity, banana	123-86-4	$C_6H_{12}O_2$	0.0176±0.00^{a}	0.0433±0.01^{ab}	0.0898±0.01^{bc}	0.1828±0.03^{d}	0.2605±0.01^{e}	0.1193±0.02^{cd}	<1	<1	2	3	4	2
13	Ethyl 2-methylbutyrate	0.000 013	Fruity	7452-79-1	$C_7H_{14}O_2$	0.0083±0.00^{b}	0.0013±0.00^{a}	0.0003±0.00^{a}	0.0013±0.00^{a}	0.0016±0.00^{a}	ND	638	100	23	100	123	-
14	Isoamyl acetate	0.000 15	Sweet, fruity, banana, solvent	123-92-2	$C_7H_{14}O_2$	0.0154±0.00^{b}	0.0019±0.00^{a}	0.0023±0.00^{a}	0.0015±0.00^{a}	0.0015±0.00^{a}	0.0015±0.00^{a}	103	13	15	10	10	10
15	Hexyl acetate	0.115	Fruity, green, apple, banana, sweet	142-92-7	$C_8H_{16}O_2$	0.0521±0.01^{bc}	0.0354±0.01^{ab}	0.0158±0.00^{a}	0.0251±0.00^{ab}	0.0691±0.00^{c}	0.0476±0.01^{bc}	<1	<1	<1	<1	<1	<1

16	Octanolactone	0.2	Fruity	698-76-0	$C_8H_{14}O_2$	0.0024±0.00^{ab}	0.0014±0.00^{a}	0.0058±0.00^{ab}	0.0098±0.00^{b}	0.0447±0.00^{d}	0.0228±0.00^{c}	<1	<1	<1	<1	<1	<1
17	Hexyl 2-methylbutyrate	0.022	Fruity	10032-15-2	$C_{11}H_{22}O_2$	0.0036±0.00^{b}	0.0012±0.00^{a}	0.001±0.00^{a}	0.0006±0.00^{a}	0.0005±0.00^{a}	0.0014±0.00^{a}	<1	<1	<1	<1	<1	<1
	Hydrocarbons																
18	Pentane	-	-	109-66-0	C_5H_{12}	1.1243±0.09^{bc}	0.7744±0.38^{abc}	0.3336±0.25^{ab}	0.5946±0.36^{abc}	1.4161±0.05^{c}	0.0365±0.02^{a}	-	-	-	-	-	-
19	Ethylbenzene	2.20525	-	100-41-4	C_8H_{10}	0.0448±0.01^{b}	0.0037±0.00^{a}	0.001±0.00^{a}	0.0039±0.00^{a}	0.0042±0.00^{a}	0.0003±0.00^{a}	<1	<1	<1	<1	<1	<1
20	p-Xylene	1	Plastic	106-42-3	C_8H_{10}	0.0737±0.01^{b}	0.0072±0.00^{a}	0.0033±0.00^{a}	0.008±0.00^{a}	0.0083±0.00^{a}	0.0012±0.00^{a}	<1	<1	<1	<1	<1	<1
21	Styrene	0.065	Sweet, balsam, floral, plastic	100-42-5	C_8H_8	0.2365±0.03^{b}	0.0235±0.01^{a}	0.0087±0.01^{a}	0.0209±0.01^{a}	0.0512±0.00^{a}	0.0084±0.00^{a}	4	<1	<1	<1	<1	<1
22	Nonane	10	-	111-84-2	C_9H_{20}	0.003±0.00^{a}	0.0015±0.00^{a}	0.0025±0.00^{a}	0.0053±0.00^{a}	0.0077±0.00^{a}	0.0054±0.00^{a}	<1	<1	<1	<1	<1	<1
23	Decane	10	-	124-18-5	$C_{10}H_{22}$	0.0398±0.00^{a}	0.0327±0.01^{ab}	0.0351±0.01^{ab}	0.0141±0.00^{a}	0.0392±0.00^{b}	0.0153±0.00^{a}	<1	<1	<1	<1	<1	<1
24	1-Methylethyl-Benzene	0.07	-	98-82-8	C_9H_{12}	0.0094±0.00^{a}	0.0077±0.00^{a}	0.0112±0.00^{ab}	0.0097±0.00^{a}	0.0195±0.00^{b}	0.0114±0.00^{ab}	<1	<1	<1	<1	<1	<1
25	o-Cymene		Citrus	527-84-4	$C_{10}H_{14}$	0.0087±0.00^{b}	0.0058±0.00^{ab}	0.0088±0.00^{b}	0.0099±0.00^{b}	0.0155±0.00^{c}	0.0038±0.00^{a}	-	-	-	-	-	-
26	p-Cymene	0.00501	Citrus	99-87-6	$C_{10}H_{14}$	0.0106±0.00^{b}	0.0065±0.00^{ab}	0.01±0.00^{b}	0.0116±0.00^{bc}	0.0171±0.00^{c}	0.0035±0.00^{a}	2	1	2	2	3	<1
27	Limonene	0.2	Lemon, citrus	138-86-3	$C_{10}H_{16}$	0.0119±0.00^{b}	0.0049±0.00^{a}	0.0056±0.00^{a}	0.0067±0.00^{a}	0.0179±0.00^{c}	0.0054±0.00^{a}	<1	<1	<1	<1	<1	<1
28	Heptane	50	-	142-82-5	C_7H_{16}	0.0156±0.00^{a}	0.0143±0.00^{a}	0.0204±0.01^{ab}	0.0135±0.00^{a}	0.0448±0.01^{b}	0.0157±0.00^{a}	<1	<1	<1	<1	<1	<1
29	Undecane	10	-	1120-21-4	$C_{11}H_{24}$	0.0221±0.00^{a}	0.0171±0.00^{a}	0.0265±0.01^{a}	0.016±0.00^{a}	0.0333±0.00^{a}	0.0278±0.00^{a}	<1	<1	<1	<1	<1	<1
30	Dodecane	10	-	112-40-3	$C_{12}H_{26}$	0.0222±0.00^{ab}	0.0145±0.00^{a}	0.0204±0.00^{ab}	0.0145±0.00^{a}	0.0321±0.00^{b}	0.0304±0.01^{b}	<1	<1	<1	<1	<1	<1
31	Tridecane		-	629-50-5	$C_{13}H_{28}$	0.004±0.00^{ab}	0.0031±0.00^{a}	0.0045±0.00^{ab}	0.0031±0.00^{a}	0.008±0.00^{c}	0.0063±0.00^{bc}						
	Aldehydes																
32	Isobutyraldehyde	0.0015	Fresh, aldehydic, floral, green	78-84-2	C_4H_8O	0.8988±0.10^{ab}	0.6502±0.32^{ab}	0.2576±0.20^{ab}	0.5103±0.40^{ab}	1.1364±0.06^{b}	0.0072±0.00^{a}	599	433	172	340	758	5
33	3-Methylbutanal	0.0011	Ethereal, aldehydic,	590-86-3	$C_5H_{10}O$	0.0029±0.00^{ab}	0.0034±0.00^{ab}	0.0039±0.00^{ab}	0.0061±0.00^{bc}	0.0084±0.00^{c}	0.0015±0.00^{a}	3	3	4	6	8	1

			chocolate, peach, fatty													
34	Pentanal	0.012	Fermented, bready, fruity, nutty, berry	110-62-3	$C_5H_{10}O$	0.0059±0.00^{a}	0.0031±0.00^{a}	0.0139±0.00^{ab}	0.0294±0.01^{bc}	0.0449±0.00^{c}	0.0279±0.00^{b}	<1	<1	1	2	4	2
35	Hexanal	0.005	Fresh, green, fatty, aldehydic, grass, leafy, fruity, sweaty	66-25-1	$C_6H_{12}O$	0.1919±0.03^{bc}	0.0435±0.01^{a}	0.0898±0.01^{a}	0.1823±0.03^{bc}	0.2607±0.01^{c}	0.1193±0.02^{ab}	38	9	18	36	52	24
36	2-Furaldehyde	9.562	Sweet, woody, almond, fragrant, baked, bread	98-01-1	$C_5H_4O_2$	0.021±0.00^{a}	0.037±0.01^{a}	0.1334±0.07^{ab}	0.2274±0.01^{ab}	0.4706±0.08^{c}	0.2648±0.07^{bc}	<1	<1	<1	<1	<1	<1
37	Heptanal	0.0028	Fresh, aldehydic, fatty, green, herbal, wine-lee, ozone	111-71-7	$C_7H_{14}O$	0.0039±0.00^{a}	0.0015±0.00^{a}	0.0121±0.00^{ab}	0.0229±0.00^{bc}	0.0275±0.00^{c}	0.0122±0.00^{ab}	1	<1	4	8	10	4
38	5-Methylfurfural	1.11	Almond, caramel, spicy	620-02-0	$C_6H_6O_2$	0.0111±0.00^{a}	0.0064±0.00^{a}	0.1266±0.06^{a}	0.1135±0.00^{a}	0.3877±0.03^{b}	0.043±0.01^{a}	<1	<1	<1	<1	<1	<1
39	1-Phenyl-1,2-propandione	0.75089	Strong, sharp, sweet, bitter, almond, cherry	100-52-7	$C_9H_8O_2$	0.0184±0.00^{a}	0.0118±0.00^{a}	0.0267±0.01^{a}	0.0334±0.00^{ab}	0.1058±0.01^{c}	0.0583±0.01^{b}	<1	<1	<1	<1	<1	<1
40	Octanal	0.000 587	Aldehydic, waxy, citrus, orange, peel, green, herbal, fresh, fatty	124-13-0	$C_8H_{16}O$	0.008±0.00^{ab}	0.004±0.00^{a}	0.0126±0.00^{abc}	0.0182±0.00^{bc}	0.07±0.00^{d}	0.024±0.00^{c}	14	7	21	31	119	41
41	Benzeneacetaldehyde	0.0063	Berry, geranium, honey, nut, pungent,floral	122-78-1	C_8H_8O	0.0046±0.00^{c}	0.0008±0.00^{ab}	0.0005±0.00^{a}	0.0013±0.00^{ab}	0.0025±0.00^{b}	0.0007±0.00^{ab}	<1	<1	<1	<1	<1	<1
42	(E)-2-Octenal	0.003	Fresh, cucumber, fatty, green, herbal, banana, waxy, leaf	2548-87-0	$C_8H_{14}O$	0.0085±0.00^{a}	0.0015±0.00^{a}	0.0022±0.00^{a}	0.0136±0.01^{a}	0.0101±0.00^{a}	0.0063±0.00^{a}	3	<1	<1	5	3	2
43	Nonanal	0.0011	Waxy, aldehydic, rose, fresh, orris, orange, peel, fatty, peely	124-19-6	$C_9H_{18}O$	0.0275±0.00^{a}	0.0106±0.00^{a}	0.0383±0.01^{a}	0.0495±0.01^{a}	0.2198±0.03^{b}	0.1854±0.05^{b}	25	10	35	45	200	169
44	(Z)-2-Decenal	-	Tallow	2497-25-8	$C_{10}H_{18}O$	0.0025±0.00^{a}	0.0028±0.00^{ab}	0.0024±0.00^{c}	0.0006±0.00^{bc}	0.0033±0.00^{d}	0.0038±0.00^{bc}	-	-	-	-	-	-

45	(E)-2,4-Decadienal	0.000 3	Fried	2363-88-4	$C_{10}H_{16}O$	0.0026±0.00^{ab}	0.0014±0.00^{a}	0.0025±0.00^{ab}	0.0019±0.00^{ab}	0.0055±0.00^{bc}	0.0066±0.00^{c}	9	5	8	6	18	22
46	(E, E)-2,4-Decadienal	0.000 027	Earthy, fried, oily	25152-84-5	$C_{10}H_{16}O$	0.0008±0.00^{a}	0.0003±0.00^{a}	0.0016±0.00^{a}	0.0023±0.00^{a}	0.0118±0.00^{b}	0.0167±0.00^{b}	30	11	59	85	437	619
	Ketones																
47	Acetoin	0.014	Sweet, buttery, creamy, dairy, milky, fatty	513-86-0	$C_4H_8O_2$	0.0026±0.00^{ab}	0.0017±0.00^{a}	0.0323±0.00^{a}	0.0072±0.00^{a}	0.0801±0.00^{a}	0.0047±0.00^{a}	<1	<1	2	<1	6	<1
48	Acetone	0.832	Solvent, ethereal, apple, pear	67-64-1	C_3H_6O	0.0004±0.00^{a}	0.013±0.00^{a}	0.2184±0.09^{b}	0.0707±0.01^{a}	0.3962±0.04^{c}	0.0077±0.00^{a}	<1	<1	<1	<1	<1	<1
49	2-Heptanone	0.14	Fruity, spicy, sweet, herbal, coconut, woody	110-43-0	$C_7H_{14}O$	0.0074±0.00^{a}	0.0041±0.00^{a}	0.0293±0.00^{b}	0.0507±0.00^{ab}	0.0972±0.00^{c}	0.0244±0.00^{a}	<1	<1	<1	<1	<1	<1
50	2-Octanone	0.0502	Herbal	111-13-7	$C_8H_{16}O$	0.0037±0.00^{a}	0.0035±0.00^{a}	0.0194±0.00^{b}	0.027±0.00^{c}	0.062±0.00^{d}	0.0139±0.00^{b}	<1	<1	<1	<1	1	<1
51	N-Methylpyrrolidone	-	-	872-50-4	C_5H_9NO	0.0105±0.00^{a}	0.0109±0.00^{a}	0.021±0.00^{c}	0.0233±0.00^{d}	0.1278±0.01^{e}	0.0489±0.01^{b}	-	-	-	-	-	-
52	4-hydroxy-2,5-Dimethyl-3(2H)-furanone	0.0223	Sweet, soap, bread	3658-77-3	$C_6H_8O_3$	0.005±0.00^{a}	0.0132±0.01^{a}	0.0305±0.01^{a}	0.0507±0.00^{ab}	0.0236±0.01^{c}	0.0109±0.00^{b}	<1	<1	1	2	1	<1
53	3-Hydroxy-2-methyl-4H-pyran-4-one	1.24	-	118-71-8	$C_6H_6O_3$	0.0021±0.00^{a}	0.0015±0.00^{a}	0.0047±0.00^{ab}	0.0025±0.00^{b}	0.0121±0.00^{ab}	0.0028±0.00^{a}	<1	<1	<1	<1	<1	<1
54	3-Nonen-2-one	0.8	Fruity, berry, fatty, oily, ketonic, weedy, spicy, licorice	14309-57-0	$C_9H_{16}O$	0.0012±0.00^{a}	0.0144±0.01^{a}	0.0068±0.00^{a}	0.0102±0.00^{a}	0.0231±0.00^{b}	0.0817±0.05^{a}	<1	<1	<1	<1	<1	<1
55	2,3-dihydro-3,5-dihydroxy-6-methyl-4H-Pyran-4-one	35	-	28564-83-2	$C_6H_8O_4$	0.0005±0.00^{a}	0.0014±0.00^{a}	0.0208±0.01^{a}	0.0381±0.01^{a}	0.2438±0.02^{a}	0.1358±0.03^{a}	<1	<1	<1	<1	<1	<1
56	2-Decanone	0.0083	-	693-54-9	$C_{10}H_{20}O$	0.0023±0.00^{a}	0.0013±0.00^{a}	0.0056±0.00^{a}	0.0063±0.00^{a}	0.029±0.00^{c}	0.0129±0.00^{b}	<1	<1	<1	<1	3	2
	Pyrazines																
57	Pyrazine	300	Roasted	290-37-9	$C_4H_4N_2$	0.001±0.00^{a}	0.0017±0.00^{a}	0.0108±0.00^{a}	0.0122±0.00^{a}	0.0218±0.00^{c}	0.0034±0.00^{b}	<1	<1	<1	<1	<1	<1
58	2-Methylpyrazine	60	Nutty, meaty, roasted	109-08-0	$C_5H_6N_2$	0.0836±0.01^{a}	0.0688±0.02^{a}	0.2586±0.08^{b}	0.2364±0.01^{b}	0.6708±0.03^{c}	0.144±0.03^{a}	<1	<1	<1	<1	<1	<1
59	2,5-Dimethylpyrazine	1.75	Roasted, nutty, popcorn	123-32-0	$C_6H_8N_2$	0.4802±0.05^{a}	0.3186±0.08^{a}	0.7016±0.18^{b}	0.9698±0.03^{ab}	2.7837±0.1^{c}	0.7895±0.17^{ab}	<1	<1	<1	<1	2	<1
60	2-Ethyl-6-Methylpyrazine	0.04	Roasted, baked potato	13925-03-6	$C_7H_{10}N_2$	0.0125±0.00^{ab}	0.0078±0.00^{a}	0.0259±0.01^{ab}	0.0324±0.00^{b}	0.1012±0.00^{c}	0.0213±0.00^{ab}	<1	<1	<1	<1	3	<1

61	2,3,5-Trimethylpyrazine	0.35012	Roasted, nutty	14667-55-1	$C_7H_{10}N_2$	0.1904±0.02^{ab}	0.1191±0.03^{a}	0.1909±0.05^{ab}	0.3026±0.01^{b}	0.7995±0.03^{c}	0.2317±0.05^{ab}	<1	<1	<1	<1	2	<1
62	3-Ethyl-2,5-Dimethylpyrazine	0.0086	Roasted, nutty	13360-65-1	$C_8H_{12}N_2$	0.0615±0.01^{ab}	0.0326±0.01^{a}	0.0547±0.02^{ab}	0.1023±0.00^{b}	0.2721±0.01^{c}	0.0859±0.02^{ab}	7	4	6	12	32	10
63	2-Ethyl-3,5-Dimethylpyrazine	0.000 04	Nutty, roasted, sweet	13925-07-0	$C_8H_{12}N_2$	0.0062±0.00^{ab}	0.0038±0.00^{a}	0.006±0.00^{ab}	0.011±0.00^{b}	0.0348±0.00^{c}	0.0104±0.00^{ab}	155	95	150	275	870	260
64	2,6-Diethylpyrazine	0.006	Roasted, nutty, sweet	13067-27-1	$C_8H_{12}N_2$	0.0076±0.00^{a}	0.0043±0.00^{a}	0.0047±0.00^{ab}	0.0109±0.00^{a}	0.0324±0.00^{b}	0.0094±0.00^{a}	1	<1	<1	2	5	2
65	2,3-Dimethyl-5-Ethylpyrazine	0.53	Nutty, roasted	15707-34-3	$C_8H_{12}N_2$	0.0076±0.00^{ab}	0.0043±0.00^{a}	0.0047±0.00^{ab}	0.0109±0.00^{b}	0.0324±0.00^{c}	0.0094±0.00^{ab}	<1	<1	<1	<1	<1	<1
66	2,3-Diethyl-5-Methylpyrazine	0.000 031	Raw peanut, potato	18138-04-0	$C_9H_{14}N_2$	0.0009±0.00^{ab}	0.0008±0.00^{a}	0.0013±0.00^{ab}	0.0027±0.00^{b}	0.0068±0.00^{c}	0.0021±0.00^{ab}	29	26	42	87	219	68
	Furan																
67	2-Acetylfuran	15.0252	Popcorn, sweet, coffee	1192-62-7	$C_6H_6O_2$	0.0036±0.00^{a}	0.0024±0.00^{a}	0.0133±0.00^{ab}	0.0124±0.00^{b}	0.0464±0.00^{c}	0.011±0.00^{ab}	<1	<1	<1	<1	<1	<1
68	2-Pentylfuran	0.0058	Fruity, green, earthy, beany, vegetable, metallic	3777-69-3	$C_9H_{14}O$	0.0079±0.00^{a}	0.0029±0.00^{a}	0.0214±0.01^{a}	0.0259±0.00^{a}	0.1252±0.00^{b}	0.0349±0.01^{a}	1	<1	4	4	22	6
	Acids																
69	Acetic acid	99	Vinegar	64-19-7	$C_2H_4O_2$	0.0286±0.01^{ab}	0.2053±0.08^{a}	0.3308±0.14^{bc}	0.4503±0.06^{c}	0.988±0.05^{d}	0.1477±0.03^{c}	<1	<1	<1	<1	<1	<1
70	2-Methylbutanoic acid	0.5	Pungent, acid, roquefort, cheese	116-53-0	$C_5H_{10}O_2$	0.002±0.00^{a}	0.0019±0.00^{ab}	0.0149±0.00^{ab}	0.0197±0.00^{b}	0.0358±0.00^{c}	0.0025±0.00^{ab}	<1	<1	<1	<1	<1	<1
	Others																
71	1-Methylpyrrolidine	0.0167	-	120-94-5	$C_5H_{11}N$	0.0002±0.00^{a}	0.0031±0.00^{a}	0.0072±0.00^{b}	0.0039±0.00^{b}	0.0245±0.01^{c}	0.0058±0.00^{a}	<1	<1	<1	<1	1	<1
72	(1H)-pyrrole	20	Sweet, nutty	109-97-7	C_4H_5N	0.0012±0.00^{a}	0.001±0.00^{a}	0.0141±0.00^{a}	0.0179±0.00^{a}	0.0317±0.00^{b}	0.0061±0.00^{a}	<1	<1	<1	<1	<1	<1
73	1-Furfurylpyrrole	0.1	-	1438-94-4	C_9H_9NO	0.0019±0.00^{a}	0.0007±0.00^{a}	0.0042±0.00^{b}	0.0034±0.00^{b}	0.0179±0.00^{c}	0.004±0.00^{a}	<1	<1	<1	<1	<1	<1
74	Pyridine	2	Medicinal, sharp	110-86-1	C_5H_5N	0.0059±0.00^{a}	0.008±0.00^{a}	0.0654±0.02^{a}	0.0483±0.00^{a}	0.1459±0.00^{b}	0.0371±0.01^{a}	<1	<1	<1	<1	<1	<1
75	2-Pentylpyridine	0.000 6	-	2294-76-0	$C_{10}H_{15}N$	0.0038±0.00^{a}	0.0011±0.00^{ab}	0.006±0.00^{c}	0.0064±0.00^{bc}	0.0554±0.01^{d}	0.0232±0.01^{abc}	6	2	10	11	92	39
76	1-Methoxy-2-Propanol	4	-	107-98-2	$C_4H_{10}O_2$	0.0009±0.00^{ab}	0.0007±0.00^{a}	0.0001±0.00^{a}	0.002±0.00^{a}	0.0008±0.00^{c}	0.0003±0.00^{b}	<1	<1	<1	<1	<1	<1
77	Trimethylamine	0.63	-	75-50-3	C_3H_9N	0.0021±0.00^{a}	0.0225±0.01^{a}	0.0515±0.01^{a}	0.0381±0.00^{a}	0.1756±0.00^{a}	0.0323±0.01^{a}	<1	<1	<1	<1	<1	<1

ND: not detected; OAVs: ratio of compound concentration to corresponding odor threshold; a & b: values with different letters in a row indicated significant differences using Duncan's multiple comparison tests ($p < 0.05$).

Table S3. The VOCs identified by the combination of olfactory time and GC-MS retention time.

NO.	RT	Compounds	Odor description	CAS	Formula
1	10.83	2-Furanmethanol	Bitter, spicy, burnt	98-00-0	$C_5H_6O_2$
2	11.14	1-Hexanol	Ethereal, fusel oil, fruity, alcoholic, sweet, green	111-27-3	$C_6H_{14}O$
3	13.29	1-Nonanol	Floral, soapy	143-08-8	$C_9H_{20}O$
4	13.53	1-Octen-3-ol	Mushroom, earthy	3391-86-4	$C_8H_{16}O$
5	15.33	1-Octanol	Soapy, oily	111-87-5	$C_8H_{18}O$
6	9.95	n-Butyl acetate	Ethereal, solvent, fruity, banana	123-86-4	$C_6H_{12}O_2$
7	10.72	Ethyl 2-methylbutyrate	Berry, sweet	7452-79-1	$C_7H_{14}O_2$
8	11.34	Isoamyl acetate	Sweet, fruity, banana, solvent	123-92-2	$C_7H_{14}O_2$
9	11.80	Styrene	Sweet, balsam, floral, plastic	100-42-5	C_8H_8
10	11.84	Nonane	-	111-84-2	C_9H_{20}
11	6.97	3-Methylbutanal	Ethereal, aldehydic, chocolate, peach, fatty	590-86-3	$C_5H_{10}O$
12	7.66	Pentanal	Fermented, bready, fruity, nutty, berry	110-62-3	$C_5H_{10}O$
13	9.71	Hexanal	Fresh, green, fatty, aldehydic, grass, leafy, fruity, sweaty	66-25-1	$C_6H_{12}O$
14	11.90	Heptanal	Fresh, aldehydic, fatty, green, herbal, wine-lee, ozone	111-71-7	$C_7H_{14}O$
15	15.00	Benzeneacetaldehyde	Berry, geranium, honey, nut, pungent,floral	122-78-1	C_8H_8O
16	15.15	2-Octenal (isomer)	Fresh, cucumber, fatty, green, herbal, banana, waxy, leaf	2548-87-0	$C_8H_{14}O$
17	16.04	Nonanal	Waxy, aldehydic, rose, fresh, orris, orange, peel, fatty, peely	124-19-6	$C_9H_{18}O$
18	19.89	(E,E)-2,4-Decadienal	Earthy, fried, oily	25152-84-5	$C_{10}H_{16}O$
19	14.79	N-Methylpyrrolidone	-	872-50-4	C_5H_9NO
20	15.39	4-hydroxy-2,5-Dimethyl-3(2H)-furanone	Sweet, soap, bread	3658-77-3	$C_6H_8O_3$
21	8.38	Pyrazine	Roasted	290-37-9	$C_4H_4N_2$
22	10.27	2-Methylpyrazine	Nutty, meaty, roasted	109-08-0	$C_5H_6N_2$
23	12.19	2,5-Dimethylpyrazine	Roasted, nutty, popcorn	123-32-0	$C_6H_8N_2$
24	14.05	2,3,5-Trimethylpyrazine	Roasted, nutty	14667-55-1	$C_7H_{10}N_2$
25	15.52	3-Ethyl-2,5-Dimethylpyrazine	roasted, nutty	13360-65-1	$C_8H_{12}N_2$
26	15.65	2-Ethyl-3,5-Dimethylpyrazine	Nutty, roasted, sweet	13925-07-0	$C_8H_{12}N_2$
27	13.78	2-Pentylfuran	Fruity, green, earthy, beany, vegetable, metallic	3777-69-3	$C_9H_{14}O$
28	10.97	2-Methylbutanoic acid	Pungent, acid, roquefort, cheese	116-53-0	$C_5H_{10}O_2$
29	7.51	1-Methylpyrrolidine	-	120-94-5	$C_5H_{11}N$
30	17.52	1-Furfurylpyrrole	-	1438-94-4	C_9H_9NO
31	17.82	2-Pentylpyridine	-	2294-76-0	$C_{10}H_{15}N$
